# A human iPSC-derived kidney–liver organ-on-a-chip platform for modeling inter-organ crosstalk

**DOI:** 10.1039/d6lc00362a

**Published:** 2026-08-13

**Authors:** Lenya de Brouwer, Victoria Pozo Garcia, Mostafa Kiamehr, Elisabeth Naderlinger, Tuğçe S. Çobanoğlu, Beren Ataç Wagegg, Eva-Maria Dehne, Catherine Verfaillie, Sofia Moco, Paul Jennings, Anja Wilmes

**Affiliations:** a Department of Chemistry and Pharmaceutical Sciences, Vrije Universiteit Amsterdam Amsterdam the Netherlands a.wilmes@vu.nl; b Amsterdam Institute of Molecular and Life Sciences (AIMMS), Vrije Universiteit Amsterdam Amsterdam Netherlands; c Department of Development and Regeneration, Stem Cell Institute, KU Leuven B-3000 Leuven Belgium; d TissUse GmbH Berlin Germany

## Abstract

The liver and kidneys are essential for drug metabolism and clearance and are highly sensitive to toxicity. Most *in vitro* models assess these organs in isolation, limiting the study of inter-organ interactions. Here, an induced pluripotent stem cell (iPSC)-derived kidney–liver organ-on-a-chip model was developed that co-cultured hepatocyte-like cell (HLC) organobodies and proximal tubular-like cells (PTL) under microfluidic flow. In parallel, a PTL–HepaRG spheroid co-culture model was established to investigate bioactivation of the prodrug ifosfamide. Co-culture induced distinct transcriptional responses in PTL and HLC, as revealed by gene set enrichment analysis. In PTL, co-culture with HepaRG enriched signaling and cell polarity pathways, while co-culture with HLC enhanced metabolic and transport-related programs. In HLC, co-culture with PTL affected intermediary metabolism, lipid processing, redox regulation, and plasma protein synthesis. The PTL–HepaRG model demonstrated bioactivation of ifosfamide into metabolite chloroacetaldehyde, highlighting the utility of these systems for studying human-relevant kidney–liver interactions and drug metabolism *in vitro*. However, the concentration of chloroacetaldehyde was too low to cause adverse effects in the renal tissue in the current setup.

## Abstract 2

An iPSC-derived kidney–liver microphysiological system recapitulates kidney–liver crosstalk, resulting in enhanced maturation of both tissues and enabling the evaluation of compounds whose biological activity depends on hepatic metabolism.

## Graphical abstract

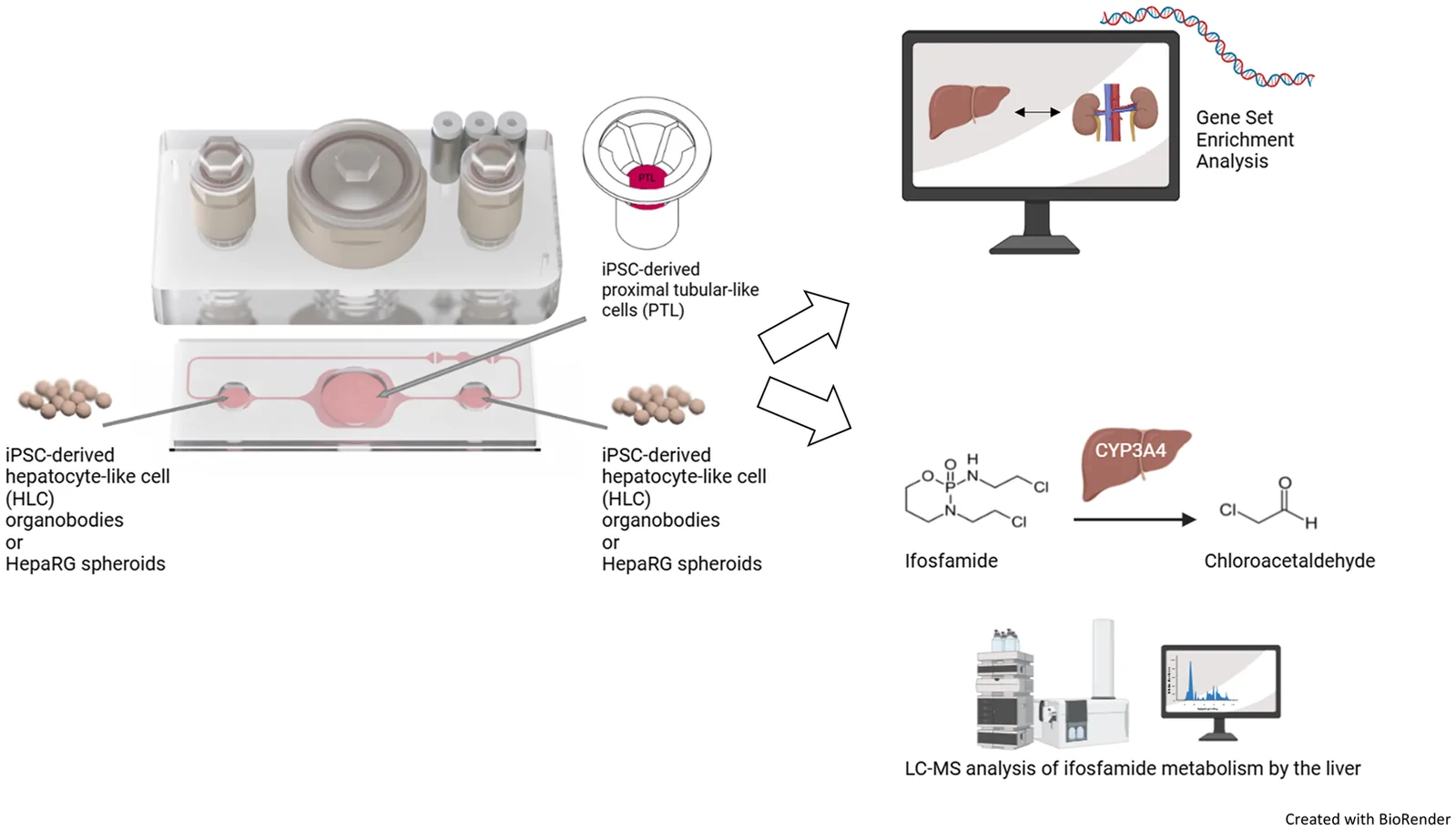

## Introduction

The liver and kidneys form an interconnected axis that is essential for maintaining homeostasis through metabolism and excretion of endogenous compounds and xenobiotics.^1^ The liver is often considered the body's primary site for drug metabolism, as it converts compounds into various metabolites *via* phase I and II enzymatic reactions.^2^ These metabolites may be eliminated *via* biliary excretion or by the kidney through glomerular filtration and tubular secretion into the urine, although they can also undergo systemic distribution and enterohepatic recirculation prior to excretion.^2,3^ The inter-organ crosstalk that takes place between the liver and kidneys is highly dynamic and bidirectional: hepatic function can influence renal clearance, whereas renal impairment can in turn alter hepatic drug metabolism and systemic homeostasis.^1^ For example, Dowling *et al.* (2003) reported that patients with end-stage kidney disease showed lower baseline hepatic CYP3A4 function than age-matched controls without kidney disease.^4^

Despite their physiological interdependence, liver and kidney *in vitro* models are often studied in isolation. The most commonly used human kidney and liver *in vitro* models consist of traditional monolayer or suspension cultures of primary cells, immortalized cell lines, or stem cell-derived cultures.^5,6^ Renal models include immortalized cell lines like RPTEC/TERT1^7^ and ciPTEC,^8^ and induced pluripotent stem cell (iPSC)-derived models like proximal tubular-like cells (PTL)^9–11^ and podocyte-like cells.^12,13^ More recently, 3D cultures like organoids, spheroids, and tubuloids derived from primary material or iPSCs have also been reported.^14–18^ Commonly used hepatic models include monolayer cultures as well as organoids and spheroids from primary human hepatocytes (PHHs), immortalized cell lines, like HepG2^19^ and HepaRG,^20^ and iPSC-derived models like hepatocyte-like cells (HLCs).^21,22^ Recently, iPSC-derived HLCs have been successfully cultured in spheroids, called organobodies.^22^ These have been extensively characterized and showed expression of several liver markers, including *ALB*, *AFP*, *HNF4a*, *NTCP*, *PEPCK1*, *G6PC* and various CYP450 enzymes. Functional characterization of HLC organobodies showed higher albumin and A1AT secretion in comparison to HLC monolayer culture and the organobodies did not show a necrotic core, based on activated caspase-3 staining.^22^

Advanced culture methods, like organ-on-a-chip (OoC) cultures, are being developed with the aim to mimic key aspects of renal and hepatic physiology, including fluid shear stress that has been shown to affect the functionality of proximal tubular cells.^5^ A review of currently available liver-on-a-chip platforms by Moradi *et al.* (2020) has shown that hepatocytes maintained function under perfusion, improving predictions of drug-induced liver injury (DILI).^23^ Similarly, kidney-on-a-chip systems have been used to successfully predict nephrotoxic responses.^24^ Next to single-organ-on-a-chip platforms, multi-organ-on-a-chip platforms are emerging, which enable the study of inter-organ communication under dynamic flow. At present, only a limited number of studies have been published on multi-organ kidney-liver chips for toxicological applications. Some of these studies incorporated animal-derived cell models, for example, canine kidney cells (MDCK) on-a-chip or rat precision cut liver slices.^25,26^ Other platforms have used immortalized human cell lines or primary cells, including a HepG2–HEK293 chip and a chip combining PHH and proximal tubular cells.^27,28^ While these studies highlight the potential of interconnected kidney–liver systems to investigate inter-organ communication and metabolite-mediated toxicity, several limitations remain. Existing models that rely on immortalized cell lines often show limited metabolic competence and do not allow for the assessment of inter-individual variability across donors.^29^ Primary tissues exhibit limited long-term culture stability, often accompanied by a loss of functional phenotype over time *in vitro* as well as restricted availability.^29–31^ Additionally, findings from animal-derived models are not always directly translatable to humans.^32^ Therefore, there is still a need for human-relevant models to overcome these limitations.

iPSC-derived models represent a promising alternative cell source. iPSCs can be differentiated into multiple organ-specific cell types derived from the same donor, enabling the development of interconnected multi-organ systems with an isogenic background.^33^ In addition, the use of iPSCs enables the generation of models from a broader and more diverse donor population, increasing population relevance and possibilities for personalized medicine.^34^ Furthermore, the ability of iPSCs to expand long-term enables large-scale production of differentiated cells and supports improved experimental standardization for toxicological assessment.^35^

In this study, we developed, to our knowledge, the first fully iPSC-derived kidney–liver organ-on-a-chip platform, consisting of a co-culture of iPSC-derived HLC organobodies and PTL maintained under microfluidic flow. Using this chip-based system, we assessed how co-culture influenced cellular identity and program-level responses relative to PTL and HLC mono-cultures. To account for the reduced metabolic capacity of the iPSC-derived liver model, a complementary PTL–HepaRG co-culture system was additionally established under both static and chip culture conditions to examine metabolic activity and absorption, distribution, metabolism and excretion (ADME)-relevant processes. HepaRG spheroids were chosen to assess metabolic activity as they are a robust, accessible and cost-friendly cell model, and CYP3A4 expression and function of HepaRG and PHH have been shown to be comparable.^36,37^

## Methods

### iPSC culture

iPSC line SIGi001-A was purchased from Sigma. This line was genetically modified to overexpress transcription factors FOXA3, PROX1 and HNF1A and referred to as SIGi001-HC3x as previously described.^21,22^ SIGi001-A cells were cultured in mTeSR™1 medium (StemCell Technologies) on Geltrex-coated plates (0.0083 mg cm^−2^, Gibco) and were passaged twice a week using Versene EDTA (Gibco).^10^ The medium was refreshed daily except for the day after passaging. The short tandem repeats (STR) profile was analyzed by Eurofins (Table S1). SIGi001-HC3x cells were expanded feeder free on Matrigel (BD Biosciences) coated plates in Essential 8 Flex (Thermo Fisher Scientific) and were passaged using EDTA (1 : 1000 dilution, Life Technologies) every 4–5 days. Cells were cultured in a humidified incubator at 37 °C with 5% CO_2_ and tested negative for mycoplasma.

### PTL differentiation

SIGi001-A cells (passage 43) were differentiated into PTL using a previously described 14-day differentiation protocol with slight modifications.^9,10^ Briefly, iPSCs were detached using Accutase (Gibco) and seeded at a density of 3.5 × 10^4^ cells per cm^2^ in PTL-A medium (1 : 1 (v/v) ratio of DMEM no glucose (Gibco, 11 966–025) and Ham's F-12 Nutrient Mix (Gibco, 21 765-029), 2 mM GlutaMAX (Gibco) and 5 μg mL^−1^ insulin (Gibco), 5 μg mL^−1^ transferrin (Sigma) and 5 ng mL^−1^ sodium selenite (Sigma)) supplemented with 1 μM CHIR99021 (Abcam), 1 μM TTNPB (Sigma), and 10 μM Y-27632 dihydrochloride ROCK inhibitor (Abcam). On day 2, the medium was replaced with PTL-A medium supplemented with 1 μM TTNPB. On day 3, the medium was replaced with PTL-B+ medium supplemented with 10 ng mL^−1^ FGF9 (Gibco). PTL-B+ medium consists of PTL-B medium (PTL-A medium with 10 ng mL^−1^ EGF (Sigma), 36 ng mL^−1^ hydrocortisone (Gibco)) supplemented with 3.5 μg mL^−1^l-ascorbic acid 2-phosphate (Sigma), 3.4 pg mL^−1^ triiodo-l-thyronine (Sigma) and 25 ng mL^−1^ prostaglandin E1 (Merck). These three additional factors were modifications compared to previously published protocols where PTL-B medium was used.^9,10^ On day 6 of differentiation, the medium was replaced with PTL-B+ medium and was refreshed every 2–3 days until day 14. Cells were then frozen in freezing medium (80% PTL-A medium, 10% (v/v) FBS (Gibco, 10 270-106) and 10% (v/v) DMSO (Sigma)) as previously described and stored in liquid nitrogen until use.^10^

### HLC differentiation and organobody formation

SIGi001-HC3X cells (passage 48–55) were differentiated into HLCs as previously described.^21,22^ Briefly, iPSCs were detached with StemPro™ Accutase® Reagent (Thermo Fisher Scientific) and plated at a density of 2 × 10^5^ cells per mL in mTeSR medium (Stem Cell Technologies) supplemented with 10 μL mL^−1^ RevitaCell (Thermo Fisher Scientific). Differentiation was started the day after in liver differentiation medium (LDM; see Table S2 for composition) until day 8. From day 0 until day 2 and from day 0 to day 4, the medium was supplemented with Wnt-3a and activin A, respectively. Starting from day 4 until the end of differentiation, 5 μL mL^−1^ doxycycline was added to induce overexpression of *FOXA3*, *PROX1* and *HNF1A*. 50 ng mL^−1^ BMP4 was added from day 4 to day 8. Growth factors were purchased from Peprotech.

On day 8, HLCs were detached with TrypLE Express (Thermo Fisher Scientific) for 8 min at 37 °C. Single cells were then resuspended in LDM with 10% (v/v) FBS (Gibco, 10 270-106), counted, and centrifuged for 4 min at 300*g*. The cell pellet was resuspended in 10% (m/v) sucrose in Milli-Q water and centrifuged again. Cells were resuspended once more in 10% (m/v) sucrose in Milli-Q water to reach a final cell density of 5 × 10^4^ cells per μL. Cells were kept on ice until further use.

A gel mixture was prepared with 5% rat tail collagen I (IBIDI, 50204), 10% laminin 521 (Thermo Fisher Scientific, A29249), 65% PuraMatrix™ peptide hydrogel (v/v, Corning, 354250) and 20% of 20% (m/v) sucrose/water. The cells and gel suspension were then mixed at a 1 : 1 (v/v) ratio by pipetting gently to avoid the formation of bubbles. The mixture was kept on ice until use.

Next, 3.5 μL of the gel–cell mixture was carefully pipetted onto the surface of 6-well plates with day 8 differentiation medium supplemented with RevitaCell to form gel–cell droplets. From day 8 to day 12, the medium was supplemented with 50 ng mL^−1^ aFGF (Peprotech). 24 h after gel–cell droplet seeding, the medium was refreshed to remove RevitaCell, and cells were maintained under these conditions until day 12. DMSO was added to the medium at a concentration of 0.6% (v/v) between day 0 and day 12 and 2% (v/v) between day 12 and day 14. From days 12 to 40, LDM was supplemented with MEM Non-essential Amino Acids (MEM NEAA (100×), Gibco, final concentration: 1×) and MEM amino acid solution (50×, Gibco, final concentration 1×); 16 mL of MEM NEAA and 8 mL of MEM amino acid solution were added per 100 mL of LDM, referred to as LDM AA3 as described by Boon *et al.*^21^ From day 12 onwards, 20 ng mL^−1^ HGF (Peprotech) was added to the medium and from day 14 onwards, the medium was further supplemented with 20 g L^−1^ glycine (Sigma). The differentiation of the HLC organobodies was continued until day 40 in LDM AA3 supplemented with HGF, glycine and doxycycline, which will be referred to as “hepatocyte medium”. Each HLC organobody was composed of approximately 87 500 cells. The organobody model has been extensively characterized in mono-culture by Kiamehr *et al.*^22^

### HepaRG differentiation and spheroid formation

Human hepatoma HepaRG cells were obtained from Biopredic International (Saint Grégoire, France) as undifferentiated cells. Cells were cultured in a 10 cm^2^ dish format with William's E medium supplemented with 2 mM GlutaMAX (Gibco, 32 551-020), 1% (v/v) penicillin–streptomycin (100 U mL^−1^ and 100 μg mL^−1^, Sigma, P4333), 9% (v/v) FBS (Gibco, 10 270-106), 50 μM hydrocortisone 21-hemisuccinate (Sigma), and 5 μg mL^−1^ human insulin (Sigma), which will be referred to as HepaRG culture medium.

Cells (passage 28) were assembled into 3D spheroids as described above, with slight modifications.^22^ Briefly, cells were washed twice with PBS and detached with trypsin–EDTA (Gibco) for 3 min at 37 °C. Detached cells were added to 10 mL pre-warmed HepaRG culture medium and centrifuged for 5 min at 300*g*. Cell pellets were resuspended in 3 mL 10% (m/v) sucrose/water. After another centrifugation, cell pellets were resuspended in 10% (m/v) sucrose/water to a final density of 5 × 10^4^ cells per μL.

The cell suspension was then mixed with equal parts (v/v) of a gel mixture containing 5% rat tail collagen (IBIDI), 10% laminin (Thermo Fisher), 20% of 20% (m/v) sucrose/water (PanReac AppliChem), and 65% PuraMatrix™ peptide hydrogel (Corning). Cells blended with the gel mixture were pipetted up and down for 2 min, avoiding air bubbles. The obtained homogeneous cell–gel mixture was dispensed (3 μL per well) into a CellCarrier Spheroid ULA 96-well Microplate (Perkin Elmer) containing HepaRG culture medium. After 48 h, cells were differentiated as previously described,^37,38^ supplementing the medium with 1.7% (v/v) DMSO for a week before co-culture with PTL. Cells were maintained in HepaRG culture medium without FBS (referred to as “M3 medium”). Cells were kept in an incubator at 37 °C under a humidified atmosphere with 5% CO_2_. Each HepaRG spheroid was composed of approximately 75 000 cells.

### PTL and HLC mono- and co-culture

PTL and HLC mono- and co-cultures were grown in TissUse HUMIMIC Chip3 chips (TissUse, C3-03-07-03-PS). Chips were equilibrated for 24 h at 48 beats per minute (bpm) in an anti-clockwise direction with either PTL-B+ medium supplemented with 100 μg mL^−1^ penicillin–streptomycin (100 U mL^−1^ and 100 μg mL^−1^, Sigma) and 1 μM GW788388 hydrate (Sigma) (to be referred to as “complete PTL-B+ medium”) for PTL mono-cultures, or with hepatocyte medium supplemented with 1 μM GW788388 hydrate for HLC mono-cultures and PTL–HLC co-cultures. The effect of each medium on the co-culture partner was assessed by exposing HLC to PTL medium and PTL to HLC medium. In both cases, cells remained viable and showed no observable abnormalities (data not shown). Medium volumes for the two 96-well compartments of the chip were 600 μL each and 800 μL for the 24-well compartment.

**Fig. 1:**
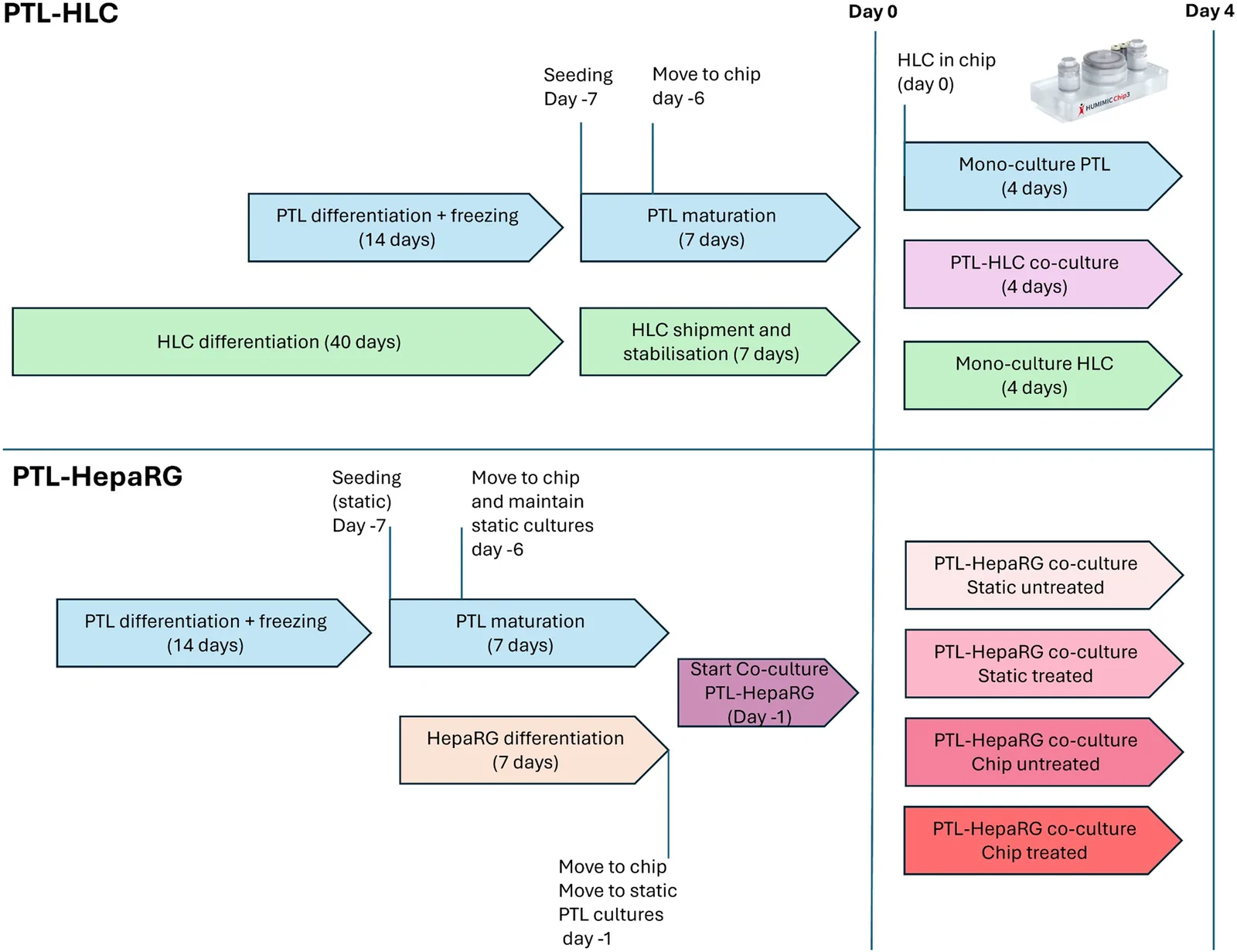
Experimental timeline of PTL, HLC, and HepaRG mono- and co-culture models. Schematic overview of the culture timeline for proximal tubular-like cell (PTL) and hepatocyte-like cell (HLC) organobody mono- and co-cultures in chips, and PTL–HepaRG static and chip co-cultures for ifosfamide exposure. Differentiation and maturation phases are indicated in blue (PTL), green (HLC), and orange (HepaRG), while co-culture conditions are shown in shades of pink. For PTL–HLC co-cultures, day 0 represents initiation of co-culture, which was maintained until day 4 alongside corresponding mono-cultures. For PTL–HepaRG co-cultures, co-culture was initiated on day −1, followed by IF treatment from day 0 to day 4.

Cryopreserved PTL (passage 1) were seeded onto Corning® cell culture Transwells® (Merck, CLS3470-48EA) with a pore size of 0.4 μm coated with 5 μg cm^−2^ collagen IV from human placenta (Merck) on day −7 at a density of 1.5 × 10^5^ cells per cm^2^ in complete PTL-B+ medium supplemented with 2% (v/v) FBS (Gibco) and 10 μM Y-27632 dihydrochloride Rho kinase inhibitor (Abcam). PTL were left to attach overnight, after which the medium was changed to complete PTL-B+ medium without FBS and Y-27632 dihydrochloride Rho kinase inhibitor. Inserts were then transferred into the 24-well compartment of the chips on day −6 (Fig. 1). After 7 days of maturation (day −7 to day 0), PTL were either mono-cultured or co-cultured with HLC organobodies. HLC organobodies were differentiated as described above and were maintained in hepatocyte medium for 7 days. Cut-off pipette tips (to avoid breaking apart of the organobodies) were then used to transfer 2 HLC organobodies into the left 96-well compartment of the chip containing hepatocyte medium supplemented with 1 μM GW788388 hydrate for both HLC mono-cultures and HLC–PTL co-cultures (co-cultures will be referred to as PTL (HLC) and HLC (PTL), representing co-cultured PTL with HLC and co-cultured HLC with PTL, respectively). During the maturation phase, PTL were fed every 2–3 days. Mono-cultures of HLC and co-cultures of HLC and PTL were fed every 2 days. During each feeding cycle, the medium was replaced fully for PTL (150 μL) or partially (600 μL) for the HLC organobodies. Cultures were kept in a humidified incubator at 37 °C with 5% CO_2_.

### Urea and albumin secretion assays

Secretion of urea and albumin into the medium was measured in mono- and co-cultured HLCs in the chips. For urea measurements, a QuantiChrom™ Urea Assay Kit (BioAssay Systems) was used according to manufacturer's instructions. Briefly, 200 μL of working reagent was added to 50 μL sample medium. Absorbance was measured at 520 nm after an incubation of 20 min at room temperature and the amount of urea per sample was calculated and expressed as ng per 10^6^ cells. Albumin secretion was measured with a Human Albumin ELISA kit from Bethyl Laboratories following the supplied protocol. In brief, samples were diluted 1 : 2 with dilution buffer to a total volume of 100 μL and were incubated in parallel with a 1 : 2 diluted albumin standard on supplied pre-coated strip plates at room temperature for 1 h. After washing 4 times, samples were incubated with 100 μL anti-albumin detection antibody for 1 h, 100 μl HRP solution for 30 min and 100 μL TBM substrate solution for 30 min, with 4 washes between every step. After TBM incubation, 100 μL stop solution was added per sample and absorbance was measured at 450 nm. The amount of albumin per sample was then extrapolated using the standard curve and expressed as ng per 10^6^ cells.

### PTL–HepaRG co-culture and ifosfamide (IF) treatment

PTL–HepaRG co-cultures were cultured in TissUse HUMIMIC Chip3 (Fig. S1). The chips were first equilibrated with M3 medium supplemented with 1 μM GW788388 hydrate for 24 h. In parallel, static PTL–HepaRG co-cultures were maintained (PTL–HepaRG co-cultures are referred to as PTL (HepaRG) and HepaRG (PTL), representing PTL co-cultured with HepaRG and HepaRG co-cultured with PTL, respectively). PTL were seeded onto 24-well cell culture inserts on day −7 and moved to the 24-well compartment of the chips on day −6. After 6 days of PTL maturation, 20 HepaRG spheroids were added to the chips; 10 spheroids were added to both the left and the right 96-well compartment. For static cultures, inserts containing PTL were first moved from the 24-well to the 6-well inserts on day −1 to ensure equal apical (150 μL) and basolateral (2 mL) volumes for both static and chip cultures. The 24-well insert was positioned into the 6-well insert by placing it in between sterile autoclaved tape. After PTL transfer, 20 HepaRG spheroids were added basolaterally into the 6-well insert on day −1. The co-cultures were left overnight, after which the HepaRG spheroids were treated with 200 μM IF (Sigma, CAS 3778-73-2) on day 0. Spheroids were re-treated with IF by means of half medium changes every 24 hours for 4 days. In parallel, cell-free static controls were treated identically to assess IF degradation and therefore potential plastic binding during treatment. PTL were cultured in PTL-B+ medium supplemented with 1 μM GW788388 hydrate; HepaRG cells were cultured in M3 medium supplemented with 1 μM GW788388 hydrate from the moment of co-culture. Cells were cultured in a humidified incubator at 37 °C with 5% CO_2._

### Transepithelial electrical resistance measurements

The transepithelial electrical resistance (TEER) was measured with an EVOM3 epithelial volt/ohm meter every 24 h during IF treatment. TEER values in Ω × cm^2^ were determined by subtracting the resistance measured in a blank, collagen IV-coated insert from the resistance values measured in inserts with cells, multiplied by the surface area of the insert.

### Immunofluorescence staining

On day 4 of culture, PTL, HLC and HepaRG mono- and co-cultures were fixed in 2% paraformaldehyde (PFA) for immunofluorescence staining. PTL were stained for megalin (R&D Systems, MAB9578, 1 : 100) and Zonula Occludens-3 (ZO-3, Cell Signalling, 3704, 1 : 1600). HLCs were stained for phosphoenolpyruvate carboxykinase (PEPCK, Santa Cruz Biotechnology, sc-271 029, 1 : 200) and cytochrome P450 3A4 (CYP3A4, BioWorld Technology, Bs90368, 1 : 100). PTL were imaged at 20× magnification with a high content imager (Perkin Elmer) and HLCs were imaged using a Nikon C2 confocal microscope with 10× magnification.

### IF and *N*-dechloroethyl ifosfamide (*N*-DCE-IF) quantification

To quantify *N*-DCE-IF, a metabolite of IF, formation over time, medium samples were taken from the apical and basolateral culture compartments after 24, 48, 72 and 96 h of treatment with IF. Aliquots (100 μL) were immediately quenched with 900 μL 80% (v/v) methanol/water (LC-MS grade, Merck) with the internal standard, 2 μM meloxicam (CAS 71125-39-8, M3935 from Merck).^37^ Control samples (cells incubated without the drug) and cell-free samples (drug stability without cells) were collected in parallel. Extracellular samples were extracted for 30 min under agitation at 4 °C. Samples were freeze-dried and dissolved in 100 μL 50% (v/v) MeOH/water. The extracts were centrifuged (5 min) and transferred into vials for LC-MS analysis.^37^

### LC-MS analysis

All solvents used during sample preparation and analysis were LC-MS grade (Merck). A previously reported LC-MS method was used.^37^ Sample acquisition was performed with a UHPLC system (Agilent 1290 UHPLC) coupled to a high-resolution time-of-flight mass spectrometer (Agilent 6230B TOF) equipped with an electrospray ionization (ESI) source. Acquisition was performed in positive ion mode. A reverse phase column (Waters XBridge BEH C18 2.1 mm internal diameter × 100 mm long, 2.5 μm particle size, and 130 Å pore size) with a guard column (Waters XBridge BEH C18 3.9 mm × 5 mm, 3.5 μm particle size, and 130 Å pore size) was used for chromatography at 40 °C column oven temperature and a flow rate of 0.2 mL min^−1^. A linear gradient was applied: 10 to 80% of B (0.1% (v/v) formic acid in acetonitrile) over 18 min, with a total run time of 24 min (A, 0.1% (v/v) formic acid in ultrapure water). A volume of 1 μL was injected for each sample, which was kept at 10 °C prior to analysis. Acquisition and data analysis were performed using MassHunter Workstation version 11.0 (Agilent). For the identification of IF (rt 9.5 min, 261.03210 *m*/*z*, within 1.5 ppm) and its metabolite *N*-DCE-IF (CAS 36 761-83-8, Sigma; *N*-dechloroethyl cyclophosphamide/*N*-dechloroethyl ifosfamide (*N*-DCE-IF); rt 3.5 min, 199.03977 *m*/*z*, within 2.6 ppm), authentic standards were run in parallel with the samples. A standard curve ranging from 0.08 μM to 20 μM (with 9 concentrations and 1 : 2 dilution steps) for both the drug and the metabolite was acquired simultaneously with the samples for compound quantification. The limit of quantification for *N*-DCE-IF was estimated at 3 nM.

### Lactate assay

To determine lactate concentrations in the culture medium, lactate assays were performed as previously described to give an indication of cellular stress.^39^ Briefly, 10 μL of supernatant which was collected during culture was mixed with lactate dehydrogenase (Sigma) and lactate reagent mix (for 10 mL reagent: 8 mL TRAM buffer, 2 mL color reagent, 3.37 mM β-NAD (Sigma, N7004/N0632) and 4 U mL^−1^ LDH (Sigma, L2500-25KU)). After a brief incubation, absorbance was measured with a CLARIOstar plate reader at 490 nm.

### Resazurin assay

To determine the cell viability after IF treatment in HepaRG mono-cultures, the resazurin reduction assay was performed as previously described.^40^ Briefly, after exposure to the compound, cells were incubated with a mixture of culture medium and resazurin (Sigma, final concentration 44 μM) for 2 h. Medium samples were then collected and the fluorescence of converted product resorufin was measured at excitation/emission of 540/590 nm with a CLARIOstar plate reader.

### Templated oligo assay with sequencing readout (TempO-Seq) transcriptomic analysis

On day 4 of culture, PTL, HLC and HepaRG mono- and co-cultures were lysed in BioSpyder 1× enhanced lysis buffer (P/N201250, lot 144 421) for the TempO-Seq Human Whole Transcriptome assay. Lysed cells were stored at −80 °C in a round-bottom 96-well plate covered with an aluminium top until shipment to BioClavis Technologies Ltd. (Glasgow, UK).

Raw gene expression counts were processed and normalized using the DESeq2 package^41^ in RStudio (2025.09.2 build 418) and R (version 4.5.1). All samples passed quality control (>15 000 raw gene counts per sample). Prior to normalization, genes represented by multiple probe identifiers were collapsed by selecting the probe with the highest mean expression across all samples. Probe identifiers were then removed, leaving only gene symbols. Datasets used for model characterization (untreated) and for the IF metabolism study (treated) were normalized independently.

For the untreated dataset, normalized gene expression values were used to assess mRNA levels of hepatocyte-, proximal tubule- and iPSC-specific markers. In addition, normalized values were used for downstream gene set-based analyses, including gene set enrichment analysis (GSEA) and gene set variation analysis (GSVA), which assess gene set responses at the group and sample level, respectively.^42,43^ Gene sets used for both GSEA and GSVA were obtained from the Molecular Signatures Database (MSigDB) collection C8 (cell-specific gene sets).^44^ For the kidney, proximal tubule (PT) gene sets PT S1–S2, S2, and S3 described by Lake *et al.* (2019) were used, which correspond to anatomically defined proximal tubule segments.^45^ For the liver, hepatocyte gene sets 1–4 derived from transcriptionally defined clusters reported by Aizarani *et al.* (2019) were used (referred to as Hep 1–4).^46^ These Hep 1–4 gene sets on MSigDB represent cluster-specific marker genes rather than predefined zonation categories but reflect a periportal-to-pericentral transcriptional gradient consistent with known metabolic zonation programs. Hep 1 was enriched for gluconeogenesis and urea cycle genes, consistent with a periportal-like metabolic phenotype. Hep 2 retained periportal–midzonal metabolic characteristics while showing broader oxidative and detoxification capacity. Hep 3 was dominated by complement, coagulation, and acute-phase response genes, consistent with hepatocyte secretory functions. In contrast, Hep 4 displayed strong xenobiotic metabolism together with enhanced transporter expression and xenobiotic-responsive regulatory pathways, consistent with pericentral detoxification programs.

For GSEA, ranked gene lists were generated from DESeq2 differential expression results using Wald test statistics as the ranking metric, which reflects the magnitude of the estimated log fold change (logFC) relative to its uncertainty. To reduce noise from lowly expressed genes, only genes with at least three normalized counts in a minimum of two samples were included in the ranked gene lists. Top 30 leading-edge driver genes were selected per gene set for each culture condition, also based on Wald statistics. For GSVA, a variance-stabilising transformation (VST) was applied to the normalized count matrix to generate expression values suitable for sample-level gene set analysis. The same expressed-gene filter (≥3 counts in ≥2 samples) was applied to define a shared gene expression universe for both GSEA and GSVA analyses, ensuring methodological consistency between approaches. Differential gene set activity in GSVA scores was assessed using the limma package.^47^ Following GSEA and GSVA, concordance between the two methods was assessed by comparing the direction and magnitude of enrichment scores derived from GSEA (normalized enrichment scores, NES) with gene set-level differential activity estimates obtained from GSVA (logFC) across corresponding gene sets and experimental comparisons. For the treated dataset, differential expression analysis was performed using DESeq2. Genes were considered differentially expressed genes (DEGs) if they met the following criteria: a DESeq2 base mean ≥5, an adjusted *p*-value ≤0.05, and an absolute log_2_ fold change (|log_2_FC|) ≥0.58, as previously reported.^48^

### Pathway analysis

Pathway analysis on the treated dataset was conducted with the gene over-representation analysis tool on ConsensusPathDB. DEGs were uploaded as HUGO Gene Nomenclature Committee (HGNC) gene symbols. Only pathways from Reactome were included for analysis after minimum overlap with the input gene list of 2. *p*-Value cutoff was set at 0.01.

### Statistical analysis

The number of replicates used for each culture setup differed per group. For transcriptomic analysis of the untreated dataset, PTL and HLC mono-cultures consisted of three and four replicates, respectively. For PTL–HLC co-cultures, three PTL and four HLC replicates were included in the analysis, as one PTL replicate was reserved for staining. For untreated PTL–HepaRG co-cultures, four PTL and five HepaRG replicates were analysed. Functional assays on HLC organobodies were performed using four biological replicates per condition. For the transcriptomic analysis of the treated dataset, four PTL and five HepaRG replicates were analysed and five biological replicates were used per condition for LC-MS analysis. Statistical analysis was performed in RStudio (2025.09.2 build 418), R (version 4.5.1) and GraphPad Prism (version 9.0.0 (121)). Data are presented as mean ± standard deviation (SD). Statistical significance was defined as *p* ≤ 0.05. Comparisons were performed using one-way or two-way ANOVA with Tukey's multiple comparisons test, as indicated in the corresponding figure legends. For transcriptomic analyses, differential gene expression was assessed using the DESeq2 Wald test with Benjamini–Hochberg false discovery rate (FDR) correction. GSEA and limma-based GSVA analyses were considered significant at an FDR ≤0.05.

## Results

### Cell-type-specific marker expression

**Fig. 2:**
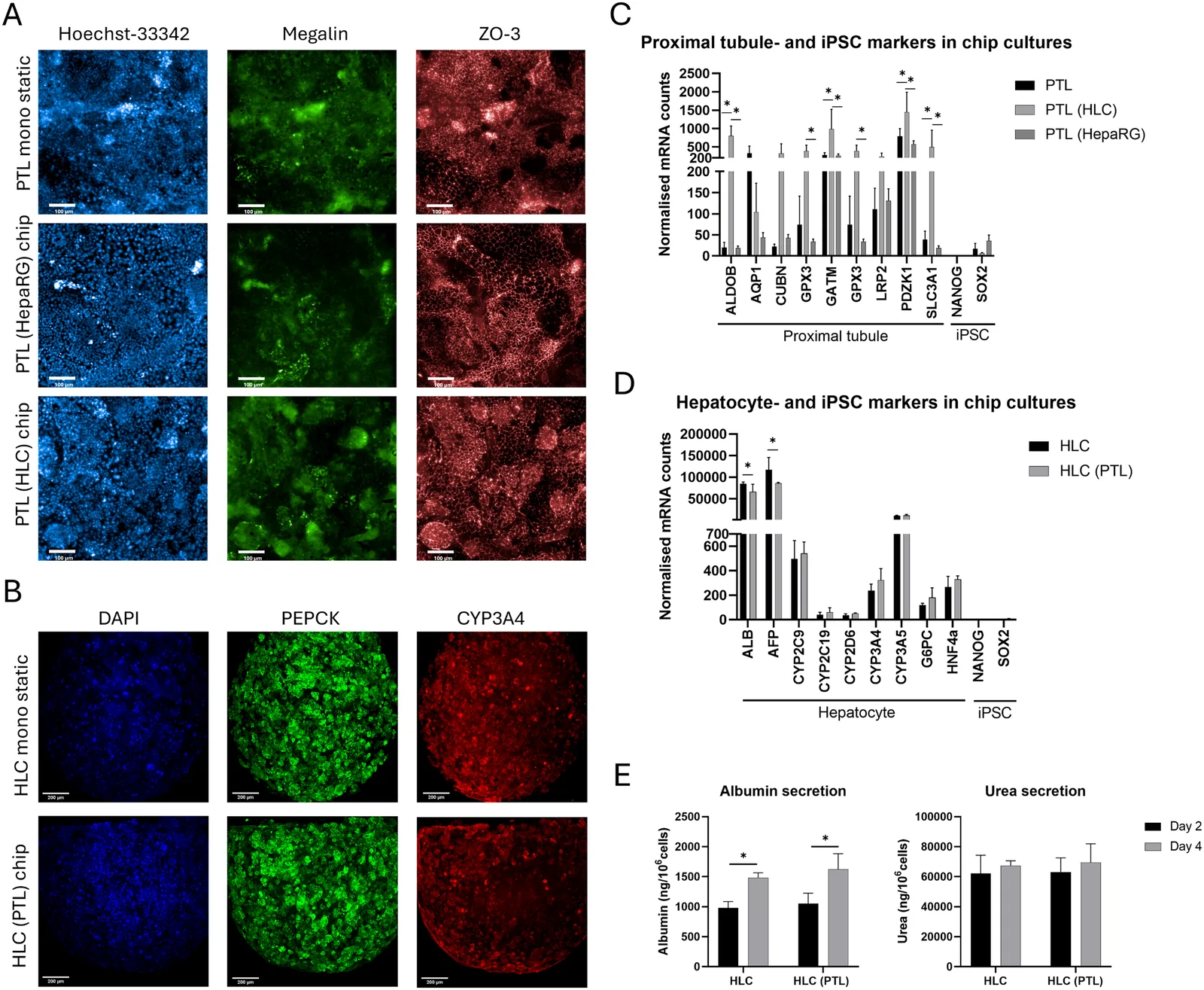
Characterization of proximal tubular-like cells (PTL) and hepatocyte-like cell (HLC) organobodies in chip co-cultures. (A) Immunofluorescence staining of PTL in chip co-cultures on day 4 of culture. Megalin is shown in green and ZO-3 in red. Nuclei were stained with Hoechst-33342. Static mono-cultures stained on day 4 are shown as controls. Images were acquired at 20× magnification (scale bar, 100 μm); representative images were selected and included in the figure. (B) Immunofluorescence staining of HLC organobodies co-cultured with PTL in chips on day 4 of culture. PEPCK is shown in green and CYP3A4 in red. Nuclei were stained with DAPI. Statically mono-cultured HLCs stained on day 4 were included as a control. Images were acquired at 10× magnification (scale bar, 200 μm); representative images were selected and included in the figure. (C) Normalized mRNA counts of selected proximal tubule and iPSC markers in PTL mono- and co-cultures on chips. Error bars represent SD (*n* = 3 (PTL and PTL (HLC)) and *n* = 4 (PTL (HepaRG)) biological replicates per condition). Statistical significance (*p* ≤ 0.05) is marked with an asterisk and was determined by two-way ANOVA with Tukey's multiple comparisons test. (D) Normalized mRNA counts of various liver and iPSC markers in HLCs mono-cultured and co-cultured with PTL on chips. Error bars represent SD (*n* = 4 biological replicates per condition). Statistical significance (*p* ≤ 0.05) is marked with an asterisk and was determined by two-way ANOVA with Tukey's multiple comparisons test. (E) Albumin (left) and urea secretion (right) in ng per 10^6^ cells over 48 h of mono-cultured HLC and co-cultured HLC with PTL in chips on day 2 and 4 of culture. Error bars represent SD (*n* = 4 biological replicates per condition). Statistical significance (*p* ≤ 0.05) was determined by two-way ANOVA with Tukey's multiple comparisons test.

Both the PTL–HepaRG and the PTL–HLC co-culture models as well as mono-cultured PTL and HLC could be maintained in culture for 4 days (Fig. 1). Phase contrast images of all models are shown in Fig. S2. Fluorescence staining of PTL in static mono-culture and in co-culture with HLC and HepaRG on chips showed expression of proximal tubule marker megalin and the tight junction marker zona occludens-3 (ZO-3) in all conditions after 4 days of culture (Fig. 2A). Similarly, staining of both static HLC mono-cultures and co-cultures with PTL on chips showed expression of liver markers phosphoenolpyruvate carboxykinase (PEPCK) and cytochrome P450 3A4 (CYP3A4) on day 4 (Fig. 2B). In addition, mRNA expression levels showed the presence of kidney- (Fig. 2C) and liver-specific (Fig. 2D) markers in mono- and co-cultured HLCs on chips, including the proximal tubular-enriched markers *LRP2* (megalin) and *CUBN* (cubilin), and hepatocyte-enriched markers *ALB*, *HNF4a* and various CYP450 genes. The absence or low expression of iPSC-specific markers *NANOG* and *SOX2* indicated successful differentiation (Fig. 2C and D). The functional capacity of HLCs was measured by urea and albumin secretion into the supernatant by mono- and co-cultured HLCs on chips on days 2 and 4 of culture (Fig. 2E). Albumin levels were significantly higher on day 4 compared to day 2 in both mono- and co-cultured HLCs, whereas urea secretion was comparable in all conditions.

### Gene set level responses introduced by co-culture

In addition, PTL mono- and co-cultures with HLC and HepaRG as well as HLC mono- and co-culture with PTL on chips were characterized through genome-wide transcriptomics using TempO-Seq. Principal component analysis (PCA) of variance-stabilized expression values showed that the main source of variance was driven by cell system and culture setup (Fig. S3).

**Fig. 3:**
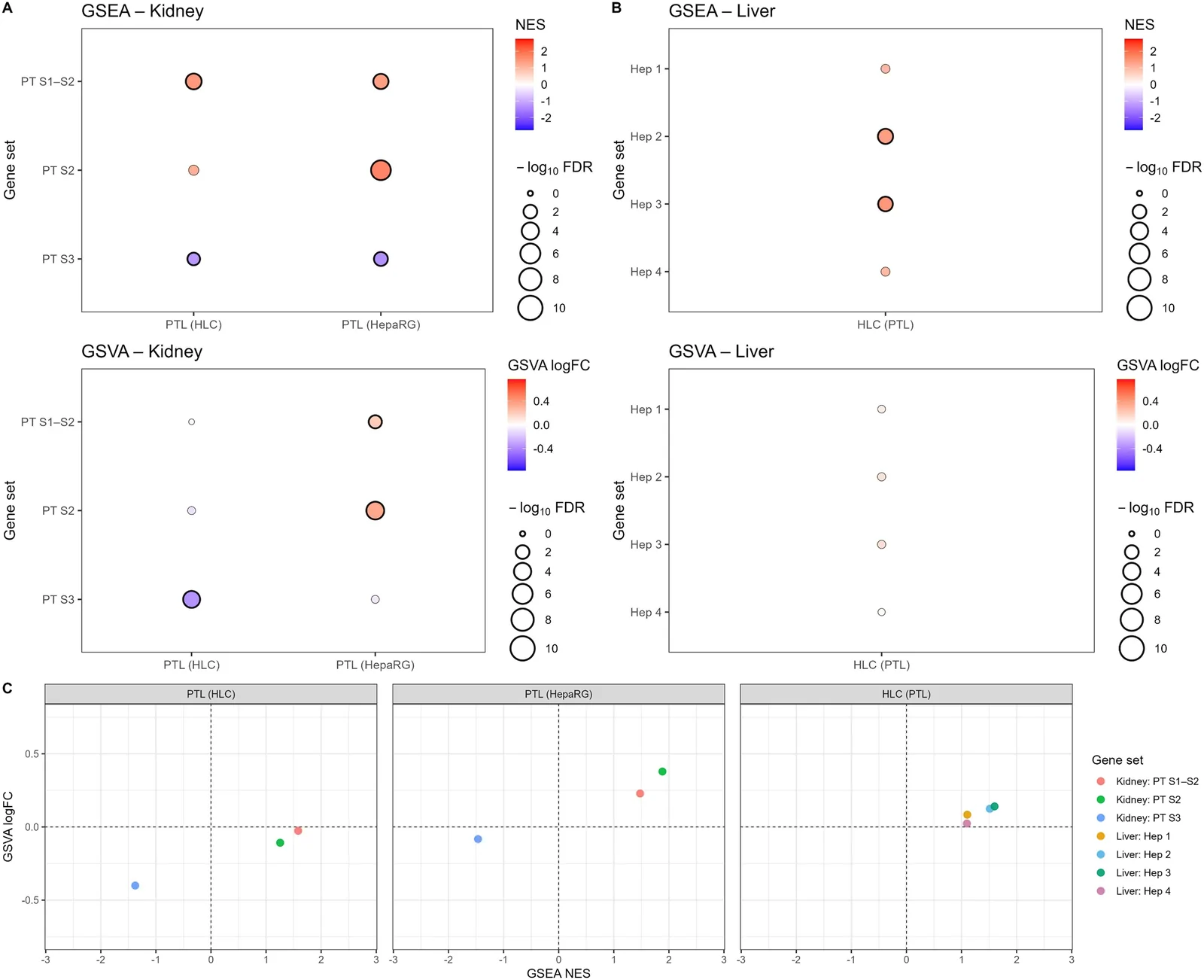
Gene set-level responses in kidney and liver co-culture models. (A) Gene set enrichment analysis (GSEA; top) and gene set variation analysis (GSVA; bottom) of proximal tubular like cells (PTL) co-cultured with hepatocyte-like cell (HLC) organobodies (PTL (HLC)) or HepaRG (PTL (HepaRG)) compared to PTL mono-cultures on day 4. GSEA plots show coordinated gene set enrichment of proximal tubule (PT) gene sets S1–S2, S2 and S3 from MSigDB based on ranked differential expression. Dot color indicates normalized enrichment score (NES), dot size represents −log_10_ false discovery rate (FDR), and thick outlines indicate statistically significant gene sets (FDR ≤0.05). GSVA enrichment scores were calculated at the sample level and subsequently compared between groups using limma. GSVA plots show group-level differential gene set activity. Dot color indicates the limma-derived log fold change (logFC) in GSVA scores, dot size represents −log_10_ FDR, and thick outlines indicate statistically significant gene sets (FDR ≤0.05). (B) GSEA and GSVA analysis of HLC organobodies co-cultured with PTL compared with mono-cultured HLC organobodies, displayed as in (A). GSEA plots show coordinated gene set enrichment of hepatocyte gene sets hepatocyte 1–4 (Hep 1–4) from MSigDB based on ranked differential expression. (C) Concordance between GSEA (NES) and GSVA (logFC), relating enrichment scores from GSEA to differences in mean GSVA gene set activity between conditions.

To assess whether co-culturing both systems altered cell-type-specific characteristics relative to mono-cultures, gene set enrichment analysis (GSEA) and gene set variation analysis (GSVA) were performed.^42,43^ Proximal tubule (PT) gene sets corresponding to specific PT segments (PT S1–S2, PT S2, PT S3) based on the study by Lake *et al.* (2019) were used for kidney analysis.^45^ GSEA of kidney cultures demonstrated significant positive enrichment of the PT S1–S2 gene set in both PTL co-cultured with HLC (PTL (HLC)) and PTL co-cultured with HepaRG (PTL (HepaRG)) compared to mono-cultured PTL (Fig. 3A). In contrast, the PT S3 gene set was significantly negatively enriched in both co-culture conditions, indicating reduced expression of PT S3 signatures in co-cultures compared to mono-cultured PTL. Interestingly, the PT S2 gene set showed stronger and statistically significant positive enrichment in PTL (HepaRG) co-cultures, whereas the enrichment was smaller and not significant in PTL (HLC) co-cultures. GSVA analysis supported these findings at the sample level (Fig. 3A). PTL (HepaRG) showed positive GSVA logFC values for PT S1–S2 and PT S2, and a negative logFC for PT S3, showing gene set activity changes consistent with GSEA results. In contrast, PTL (HLC) showed negative GSVA logFC values for all PT programs; even though some showed positive enrichment in GSEA.

Hepatocyte gene sets hepatocyte 1–4 (Hep 1–Hep 4), based on the previously described transcriptionally defined clusters,^46^ were used for liver GSEA and GSVA. Analysis of liver cultures revealed that HLC co-cultured with PTL (HLC (PTL)) exhibited higher activity of liver-specific gene sets, most prominently the Hep 1 and Hep 2 gene sets, corresponding to hepatocyte programs associated with oxidative metabolism and hepatic plasma protein synthesis and secretory functions, respectively (Fig. 3B). These programs were significantly enriched by GSEA, whereas GSVA showed concordant but non-significant increases in gene set activity (Fig. 3B).

To further evaluate the agreement between enrichment approaches, concordance analysis was performed comparing GSEA NES values with GSVA logFC values. PTL (HLC) co-cultures showed reduced directional alignment between GSEA and GSVA. While PT S1–S2 and PT S2 signatures were positively enriched in GSEA, GSVA indicated negative log fold changes for these gene sets in co-culture relative to mono-culture, as reflected in the concordance plot (Fig. 3C). This pattern suggests heterogeneous PT S1–S2 and PT S2 program responses in PTL (HLC) co-cultures, with GSEA enrichment likely driven by a subset of strongly regulated genes rather than uniform changes across the gene set. In PTL (HepaRG) co-cultures, PT S1–S2 and PT S2 gene sets showed strong directional agreement between GSEA and GSVA, with both methods indicating higher program activity in co-culture relative to mono-culture (Fig. 3C). PT S3 gene sets clustered in the negative quadrant, showing agreement that PT S3 gene sets were enriched in mono-culture rather than co-culture. Concordance analysis of liver cultures demonstrated consistent directional agreement between GSEA and GSVA for liver-specific gene sets, indicating consistency in gene set-level responses across the two different analysis approaches (Fig. 3C).

### Leading-edge genes underlying distinct program changes induced by co-culture

**Fig. 4:**
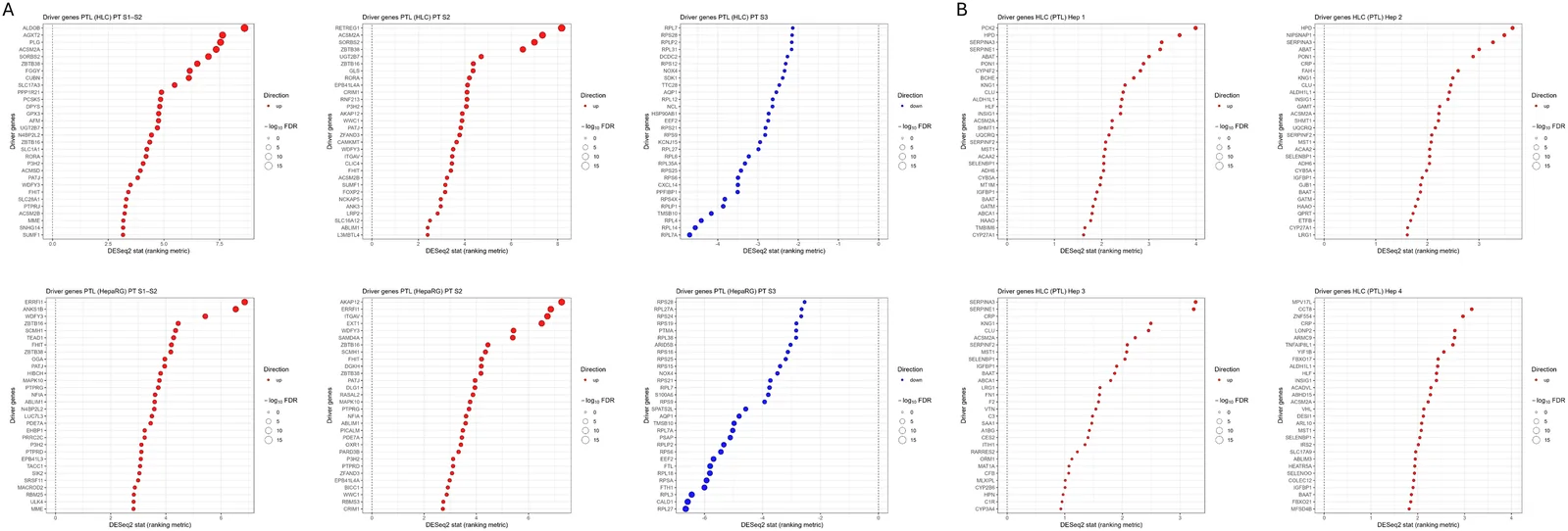
Leading-edge genes driving gene set enrichment analysis (GSEA)-identified program shifts. (A) Leading-edge genes contributing to the enrichment signal observed in GSEA for proximal tubule (PT) gene sets S1–S2 (left), PT S2 (middle), and PT S3 (right) in proximal tubular-like cells (PTL) co-cultured with hepatocyte-like cell (HLC) organobodies (PTL (HLC), top) and PT co-cultured with HepaRG (PTL (HepaRG), bottom). (B) Leading-edge genes contributing to the enrichment signal observed in GSEA for gene sets hepatocyte 1 (Hep 1, top left), hepatocyte 2 (Hep 2 (top right)), hepatocyte 3 (Hep 3 (bottom left)), and hepatocyte 4 (Hep 4 (bottom right)) signatures in HLC organobodies co-cultured with PTL (HLC (PTL)). Genes are ranked by DESeq2 Wald statistics. Dot size reflects significance (−log_10_ FDR), and color indicates direction of change (red, upregulated; blue, downregulated).

To identify the genes contributing most strongly to the GSEA-identified program shifts, leading-edge gene analysis was conducted. This revealed that despite similar directional enrichment of PT S1–S2 and negative enrichment of S3 gene sets across both PTL (HLC) and PTL (HepaRG), the driver genes of these responses differed substantially depending on the co-culture cell type (Fig. 4A). For the PT S1–S2 program, PTL (HLC) driver genes were predominantly associated with proximal tubule metabolic and transport function, including carbohydrate and lipid metabolism (*ALDOB*, *ACSM2A*), xenobiotic detoxification (*UGT2B7*), and solute reabsorption (*CUBN*, *SLC17A3*) (Fig. 4A). In contrast, PTL (HepaRG) S1–S2 drivers were involved in signaling and transcriptional regulation, including *ERRFI1*, *ZBTB16*, and *TEAD1* as well as cytoskeletal and polarity-associated genes like *PATJ* and *ABLIM1* (Fig. 4A).

In the PT S2 gene sets, PTL (HepaRG) co-cultures exhibited significant enrichment which was mainly driven by genes involved in signal transduction, transcriptional and epigenetic regulation and cell polarity, including *ERRFI1*, *MAPK10*, *PTPRG*, *NFIA* and *PARD3B* (Fig. 4A). In contrast, in PTL (HLC) the PT S2 gene set did not reach statistical significance in GSEA, indicating a less uniform S2-specific transcriptional response in this condition. Nevertheless, highest expression changes were observed in genes associated with metabolic processing, transport, and epithelial structure, such as *ACSM2A*, *GLS*, *LRP2* and *SLC16A12*, (Fig. 4A). In contrast to S1–S2 and S2 segments, PT S3 leading-edge genes in both PTL (HLC) and PTL (HepaRG) co-cultures were dominated by ribosomal, translational, and stress-associated genes, including multiple ribosomal subunits (*RPL7A*, *RPS6*), the elongation factor *EEF2* and oxidative stress-related genes such as *NOX4* and *FTH1* (Fig. 4A). All S3 driver genes exhibited negative Wald statistics, indicating coordinated downregulation of translation- and stress-associated programs in co-culture relative to mono-culture.

For the liver, leading-edge genes in the Hep 1 gene set could mainly be linked to intermediary and energy metabolism, including gluconeogenesis and amino acid metabolism (*PCK2*, *HPD*) and fatty acid metabolism (*ACSM2A*, *ACAA2*) (Fig. 4B). In addition, multiple plasma protein and protease regulator genes (*SERPINA3*, *SERPINE1*, *SERPINF2*) were identified, consistent with hepatocyte-specific secretory activity. The Hep 2 gene set showed a similar profile, with drivers linked to metabolic processing (*FAH*, *ABAT*, *ACSM2A*), cholesterol regulation (*INSIG1*), and mitochondrial metabolism (*ETFB*) (Fig. 4B). Leading edge genes for the Hep 3 program highlighted hepatic secretory and plasma proteins (*CRP*, *F2*, *C3*, *VTN*), genes involved in lipid handling and bile acid metabolism (*ACSM2A*, *BAAT*, *ABCA1*) and genes associated with xenobiotic metabolism, like *CES2*, *CYP2B6*, *CYP3A4* (Fig. 4B). The Hep 4 program was characterized by genes linked to fatty acid oxidation and metabolic regulation (*ACADVL*, *ACSM2A*), redox balance (*SELENOO*, *SELENBP1*), and protein homeostasis (*CCT8*, *FBXO17*, *FBXO21*, *VHL*) (Fig. 4B). Across the different hepatocyte gene sets, leading-edge genes were largely associated with metabolism, redox regulation, lipid processing, and plasma protein synthesis.

### Ifosfamide bioactivation in the PTL–HepaRG spheroid co-culture model

**Fig. 5:**
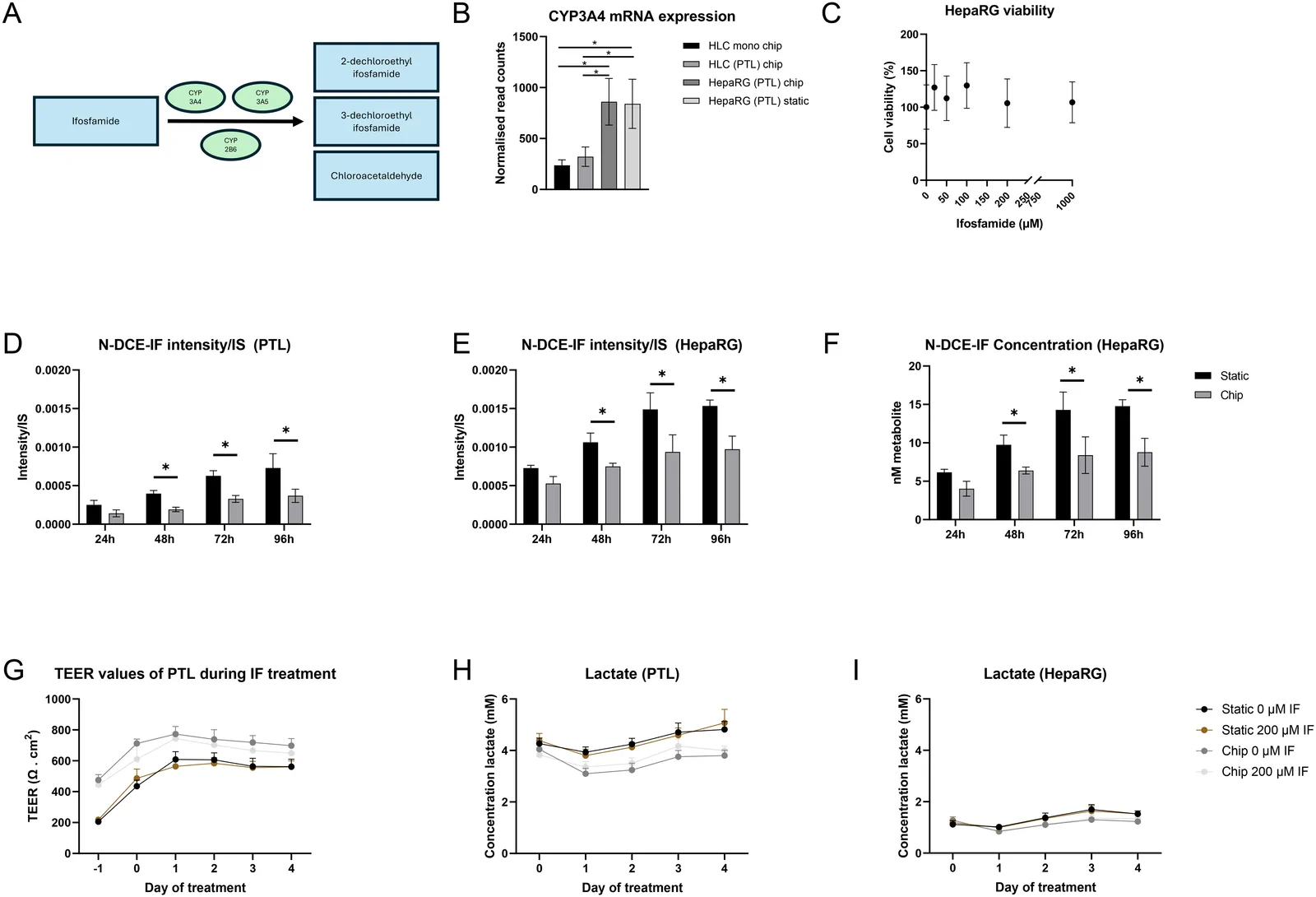
Functional and metabolic responses to ifosfamide in PTL–HepaRG co-cultures over a time course of 4 days. (A) Schematic of metabolic conversion of ifosfamide (IF) to N-dechloroethyl cyclophosphamide (*N*-DCE-IF). (B) CYP3A4 mRNA expression levels in liver mono- and co-culture models. (C) Viability of HepaRG spheroids following treatment with increasing concentrations of IF. (D and E) Intensity/internal standard (IS) ratios of *N*-DCE-IF in the apical compartment containing proximal tubular-like cells (PTL) (D) and the basolateral compartment containing HepaRG spheroids (E). (F) Concentration of *N*-DCE-IF in the basolateral compartment containing HepaRG spheroids. (G) Transepithelial electrical resistance (TEER) of PTL in co-culture with HepaRG spheroids during IF treatment. (H and I) Lactate concentrations in medium from PTL (H) and HepaRG (I) during IF treatment. Error bars represent SD (*n* = 5 biological replicates per condition ((C–I), and HepaRG (PTL) in (B)), *n* = 4 in HLC mono chip and HLC (PTL) chip in (B)). Statistical significance (*p* ≤ 0.05) was determined by one-way ANOVA (B) and two-way ANOVA (D–F) with Tukey's multiple comparisons test.

In addition to transcriptomic characterization of these models, ifosfamide (IF) was used as a model compound to assess metabolism and toxicity. IF is an anti-cancer agent known to induce nephrotoxicity in the clinic upon bioactivation into the metabolite chloroacetaldehyde (CAA).^49^ As CAA could not be measured through LC-MS directly, the surrogate *N*-dechloroethyl ifosfamide (*N*-DCE-IF) was measured instead (Fig. 5A). This metabolite of IF is also metabolized by CYP3A4 in equimolar amounts as CAA.^26^ In order to assess bioactivation of IF into *N*-DCE-IF, the PTL–HepaRG spheroids co-culture system was used in both static and chip conditions, as IF metabolism into CAA occurs primarily *via* CYP3A4, which is highly functional in HepaRG cells.^50^ This was also confirmed by transcriptomics results which showed higher CYP3A4 mRNA expression in HepaRG than in HLC cultures (Fig. 5B). Based on HepaRG viability assessments (Fig. 5C) and LC-MS detection limits, a treatment concentration of 200 μM IF was selected to maximize drug exposure while avoiding detector saturation. The metabolite *N*-DCE-IF was detected in both PTL and HepaRG compartments at all measured time points (Fig. 5D and E), indicating metabolism of IF by HepaRG spheroids. IF itself was also detected in both compartments and remained stable over time (Fig. S4). *N*-DCE-IF levels detected in the basolateral compartment containing HepaRG spheroids corresponded to nanomolar concentrations, with peak levels reaching approximately 15 nM. Values of *N*-DCE-IF differed significantly between static and chip cultures at 48, 72, and 96 h of treatment, with *N*-DCE-IF reaching higher levels in static than in chip cultures (Fig. 5F). In the apical compartment containing PTL, *N*-DCE-IF levels were below the limit of quantification (=3 nM) at all the measured time points and could therefore not be converted into concentrations. In parallel, cell free static controls were exposed to IF to determine potential plastic binding (Fig. S5). No evidence was found that IF bound to the plastic of the plate during exposure. Potential binding of IF to the PDMS of the chips was calculated to be 0.2824 (log *K*(PDSM)), suggesting weak binding of IF to PDMS would occur.^51^ TEER measurements of PTL indicated kidney cultures maintained a tight monolayer throughout IF exposure and remained comparable between the IF-treated and the control conditions in both static and chip cultures throughout the treatment period (Fig. 5G). TEER values increased from day −1 to day 1 and subsequently stabilized, with slightly higher values observed in chip cultures relative to static cultures. Values in all culture conditions indicated intact barrier integrity during treatment. Lactate concentration in the culture medium was measured as an indicator of cellular stress.^39^ Concentrations remained stable over time in both HepaRG and PTL compartments, with no significant differences between treated and untreated conditions in either static or chip cultures (Fig. 5H and I), suggesting a lack or low levels of cellular stress during treatment. Overall lactate levels were slightly higher in static cultures compared to chip cultures.

**Fig. 6:**
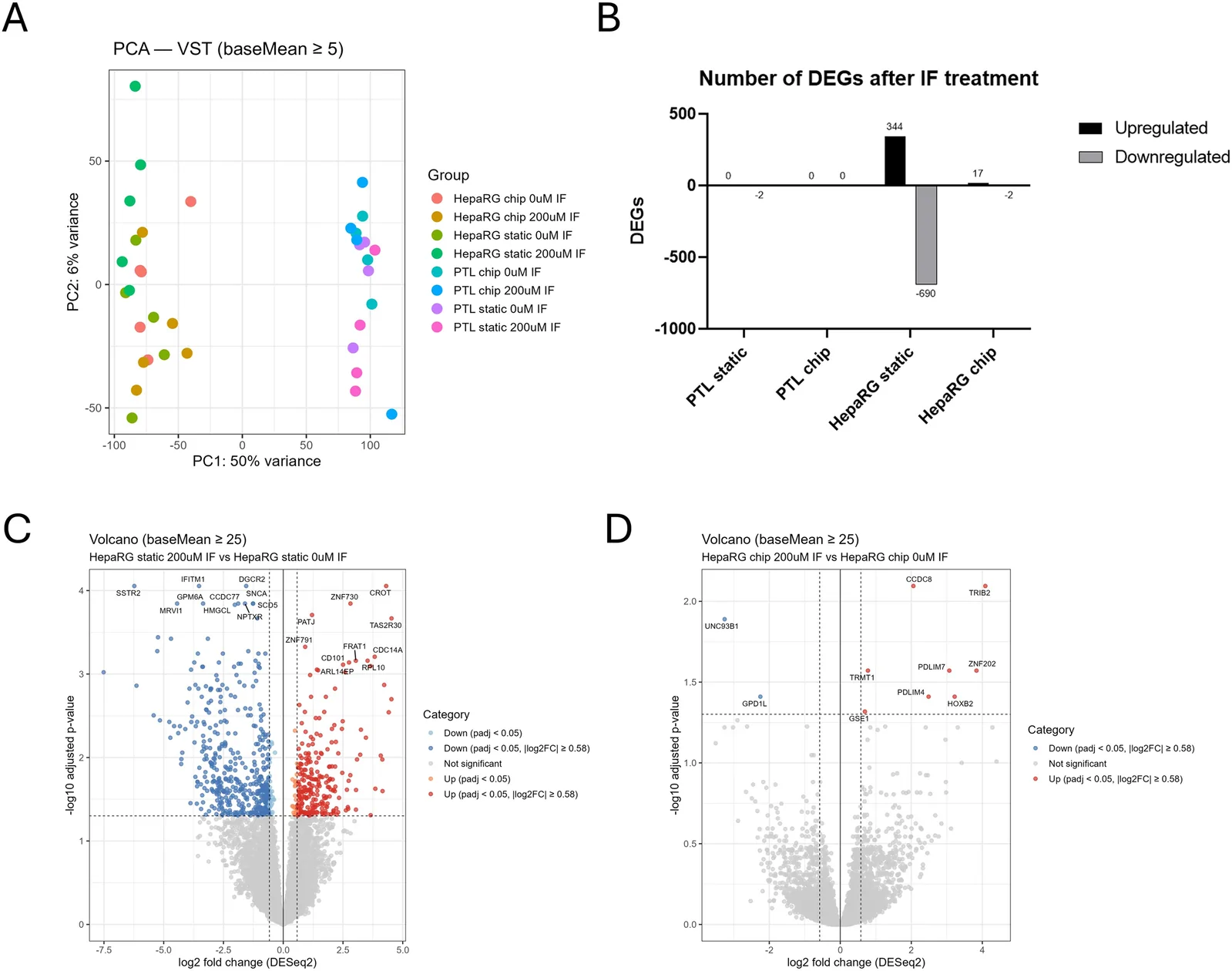
Differential gene expression following ifosfamide exposure in PTL–HepaRG co-cultures. (A) Principal component analysis (PCA) of proximal tubular-like cells (PTL) and HepaRG spheroid samples. PTL–HepaRG co-cultures were treated with 0 or 200 μM ifosfamide (IF) for 4 days and then lysed separately. (B) Number of up- and downregulated differentially expressed genes (DEGs) per condition after IF treatment (base mean ≥5, adjusted *p* value ≤0.05, |log_2_FC| ≥0.58). (C and D) Volcano plots of DEGs following IF treatment in static (C) and chip-cultured (D) HepaRG spheroids. Only genes with base mean ≥ 25 were included. The top 10 (or fewer, if applicable) most significantly up- and downregulated genes were labeled. Sample size was *n* = 5 for all conditions.

Gene expression changes in PTL–HepaRG co-cultures after 4 days of IF treatment compared to untreated controls were assessed through transcriptomics. PCA of variance-stabilized expression values showed clear separation between PTL and HepaRG samples, but no clear separation between treated *versus* untreated samples was observed (Fig. 6A). This correlated with the lack of toxicity observed based on lactate measured in the supernatant (Fig. 5H and I) and viability assays of CAA in PTL, which only showed signs of CAA-induced toxicity in PTL at concentrations above 100 μM (Fig. S6). Interestingly, differential expression analysis of the co-cultures revealed condition- and culture-dependent transcriptional responses to IF treatment. In PTL, no significantly differentially expressed genes (DEGs) were detected following 4 days of treatment under chip conditions and only 2 downregulated DEGs were identified in static cultures (Fig. 6B). In contrast, HepaRG spheroids showed a stronger transcriptional response. In the chip, 17 upregulated and 2 downregulated DEGs were detected following IF exposure. The largest response was observed in static cultures, with 344 upregulated and 690 downregulated DEGs identified for HepaRG spheroids (Fig. 6B–D). *TRIB1* was the only DEG shared after IF treatment between static and chip conditions in HepaRG spheroids. Gene and pathway analysis of upregulated genes with ConsensusPathDB showed no clear signs of activation of classical stress response pathways typically caused by IF, like apoptosis or the Nrf2 pathway.^52,53^ Interestingly, CYP3A4 and CYP2C9, which are both involved in the conversion of IF into *N*-DCE-IF, were among the DEGs for IF-treated HepaRG compared to controls in static culture.

## Discussion

In this study, we report on a fully iPSC-derived kidney–liver organ-on-a-chip platform that enables co-culture of HLC organobodies and proximal tubular-like cells under microfluidic flow for a minimum of 4 days. Our findings demonstrate that inter-organ co-culture influences cellular identity and pathway activity compared to mono-cultures, highlighting the importance of organ–organ communication in *in vitro* models.

Relatively few kidney–liver organ-on-a-chip studies have investigated changes in gene expression levels associated with inter-organ co-culture compared to mono-culture conditions. In particular, transcriptomic analyses remain limited, as most previously reported systems primarily focused on toxicity endpoints or metabolic activity. Nevertheless, some studies have explored gene expression changes in these models.

Nguyen *et al.* (2022) compared the expression of a selection of proximal tubule-specific genes in kidney tubuloid mono-cultures and co-cultures with liver organoids on-a-chip through qPCR. They reported comparable expression levels of key proximal tubule markers and transporters, including *SLC22A6*, *SLC22A8*, *SLC22A2*, *SLC2A2*, *SLC5A2*, *CUBN* and *ABCB1*, between mono- and co-culture conditions.^54^ A similar comparison was performed between liver mono-cultures and liver–kidney co-cultures, where a modest decrease in *CYP3A4* and *CYP1A2* and increase of *ABCC2* under co-culture conditions was observed.^54^ In contrast, our findings showed *CUBN* as a driver gene for the PT S1–S2 gene set in co-cultured PTL (HLC) and *CYP3A4* as a driver gene for Hep 3 in co-cultured HLC (PTL) compared to mono-cultured HLC. Liu *et al.* (2026) compared gene expression in a kidney-only chip using spheroids from the human cancer line HK-2 with a liver–kidney co-culture system on-a-chip containing rat-derived precision-cut liver slices and HK-2 spheroids. They found that co-culture altered the baseline gene expression profile of HK-2 cells, with enrichment of genes associated with the CD40 complex and mitochondrial intermembrane space.^25^ Our findings also showed changes in gene expression levels between mono- and co-cultures. However, leading-edge analysis revealed that the genes driving the gene set responses differed substantially depending on the hepatic co-culture partner. For example, in PTL (HLC) co-cultures, the PT S1–S2 enrichment was primarily driven by genes related to metabolic processing and solute transport, indicating a more classically expected proximal tubule profile, whereas PTL (HepaRG) co-cultures showed enrichment of signaling and transcriptional regulators as well as genes associated with cytoskeletal organization and polarity. These differences suggest that while kidney–liver co-culture consistently affects proximal tubule gene expression, the nature of the response may depend on the hepatic co-culture cell type and the signals exchanged between the two compartments. However, it should be noted that the amount of HepaRG spheroids in co-cultures with PTL was substantially higher than the amount of HLC organobodies in co-cultures with PTL. Specifically, 2 HLC organobodies were used in each co-culture, whereas 20 HepaRG spheroids were used. The reason for this was to maximize CYP3A4-mediated bioactivation of IF by HepaRG, rather than characterizing the impact of the cancer cell line on the iPSC-derived PTL. Consequently, the differences in the total amount of liver cells in the different co-cultures could have contributed to the gene expression changes observed. Based on this study, it cannot be determined with certainty to what degree the observed gene changes were caused by the amount of liver cells or by biological changes induced by the distinct liver cell types within the co-cultures. However, as distinct gene sets were enriched in each co-culture condition, the observed gene expression changes are unlikely to be driven solely by differences in cell number.

A key strength of a fully iPSC-derived kidney–liver organ-on-a-chip model is the ability to generate isogenic cell types for both organs from multiple donors.^33^ This enables the development of donor-specific models and facilitates the study of inter-individual variability, providing opportunities for more personalized and population-relevant studies.^34^ In addition, the renewable nature of iPSC-derived cells allows the generation of large numbers of experimental replicates from the same genetic background.^35^ However, several limitations should also be considered. In general, iPSC-derived cell types do not always reach the functional maturity that is observed in primary cells or certain established cell lines.^55^ While the HLC organobodies showed reasonable expression and function of CYP3A4, this was higher in HepaRG spheroids. This is in line with previous findings by us and others, which generally report relatively low CYP3A4 activity or expression in various iPSC-derived HLC models in comparison to PHH and hepatocyte cell lines.^37,56,57^

Our findings showed that IF was successfully metabolized into *N*-DCE-IF by the HepaRG spheroids. *N*-DCE-IF was detected in both the PTL and the HepaRG compartments. However, this metabolite could only be quantified in the liver compartment, with peak concentrations reaching 15 nM in static cultures. In the kidney compartment, *N*-DCE-IF was detected, but quantification was not possible as levels observed were below the limit of quantification of the employed LC-MS instrument (=3 nM). This suggests that metabolite levels of CAA reaching the kidney compartment were most likely too low to induce nephrotoxicity. The lack of nephrotoxicity is likely a scaling limitation rather than evidence of biological insensitivity of the renal model, as toxicity in PTL mono-cultures was successfully induced after treatment with CAA directly (Fig. S6). Animal and clinical pharmacokinetic studies have reported circulating CAA concentrations in the low micromolar range following ifosfamide administration, with peak plasma concentrations ranging from approximately 10–45 μM depending on the species and dosage.^58,59^ This is in line with previously reported results that show treatment with CAA in the human proximal tubular cell line RPTEC/TERT1 could induce cellular stress after repeated 24 h exposure at concentrations of 10 and 35 μM.^60^ After 1, 3, and 14 days, a time- and dose-dependent increase in DEGs was observed. DEGs were mainly associated with a strong Nrf2 oxidative stress response and an induced expression of the unfolded protein response (UPR)-dependent genes was observed.^60^ On the other hand, treatment with the parent compound IF only showed effects in mM concentrations in RPTEC/TERT1 cells.^60^ In the current study, treatment with 200 μM IF resulted in a maximum measured *N*-DCE-IF concentration of approximately 15 nM in the liver compartment, consistent with low CAA-generating bioactivation that was most likely insufficient to cause nephrotoxicity. *In vivo*, a large hepatic biomass continuously generates metabolites into the circulation, whereas in the present chip format a comparatively small number of hepatic cells is distributed over a relatively large recirculating medium volume. This likely diluted metabolite output below the threshold required to elicit measurable renal stress responses. Given the fixed fluid-to-tissue volume ratio of the current chip layout, even a high increase in hepatic biomass would unlikely increase the metabolite concentration enough to show nephrotoxic effects of IF. Even increasing the hepatic biomass by 50 additional spheroids, which can be added to the 24-well compartment, would unlikely increase the metabolite concentration enough to show nephrotoxic effects for this compound.

Interestingly, our findings showed that IF treatment induced a number of DEGs in the static HepaRG spheroids, and, to a lesser extent, in chip-cultured HepaRG. However, the DEGs were not associated with changes in the Nrf2 or UPR responses that were observed in renal cells in previous studies.^60^ A possible explanation for the stronger transcriptional response in static HepaRG could be the amount of CAA formed, which was roughly 15 nM in static and approximately 9 nM in chip cultures. Interestingly, CYP3A4 and CYP2C9 expression levels were increased in the static HepaRG cultures but not in HepaRG chip cultures after treatment with IF. This could indicate a higher activity of these enzymes in static cultures, leading to more metabolites of IF being formed. Another study by Duivenvoorde *et al.* showed that CYP3A4 expression decreased once HepaRG cells were cultured in chips compared to static cultures, which could possibly explain the lower amounts of metabolite observed in the chip cultures.^36^

IF and CAA nephrotoxicity have also been examined in other liver–kidney organ-on-a-chip setups using HepaRG and HepG2 cells with MDCK canine kidney cells. CAA formation was only reported in the HepaRG and MDCK co-culture system after treatment with 50 μM IF, which was lower than the concentrations used in this study, despite the use of a similar hepatic cell source.^26^ This difference could be explained by the different chip platforms, medium volumes and culture conditions of HepaRG cells. Additionally, MDCK are canine cells which are generally associated with a more distal tubule characterization.^61^ This could potentially explain the differences observed in CAA toxicity at lower doses.

Accurately modeling drug bioactivation *in vitro* remains challenging, as enzyme activity, transport processes, and inter-organ distribution are not always fully representative of the *in vivo* situation, in particular for CYP3A4 activity.^62^ This is further complicated by the fact that iPSC-derived hepatocyte models often exhibit incomplete metabolic maturation.^63^ Further studies are needed to improve the xenobiotic metabolic capacity of iPSC-derived hepatocytes and to optimize hepatic tissue mass to generate high enough metabolite concentrations to cause nephrotoxic effects in the renal tissue. Lastly, assessment of other compounds with a different route of bioactivation could provide additional information.

## Conclusions

In conclusion, we have established a human microphysiological system combining iPSC-derived PTL with iPSC-derived HLC organobodies or HepaRG spheroids under microfluidic flow to evaluate inter-organ crosstalk and drug metabolism. While co-culture induced distinct, cell-type-specific transcriptional responses that enhanced functional markers and metabolic programs across both tissue compartments, our findings highlight critical considerations for modeling prodrug toxicity *in vitro*. Exposure to ifosfamide successfully demonstrated active hepatic bioactivation and basolateral to apical metabolite transport into the renal compartment. However, peak generated metabolite concentrations were in the low nanomolar range, whereas *in vivo* and clinical data report micromolar concentrations. Nonetheless, this platform demonstrates the utility of fully human multi-organ systems for capturing baseline organ crosstalk and active metabolite transport. Future implementations should focus on optimizing tissue-to-medium volume ratios and scaling hepatic metabolic capacity to bridge the gap between *in vitro* metabolite kinetics and *in vivo* systemic exposure.

## Author contributions

Experiment design: A. W., S. M., V. P. G., E. D., B. A. W. Execution of experiments: V. P. G., E. N., M. K., L. B. Data analysis L. B., T. S. C., V. P. G., M. K., E. N. Data visualization: L. B. Writing – original draft: L. B., A. W. Writing – review and editing: L. B., A. W., P. J., V. P. G., S. M., T. S. C., M. K., C. V., E. N., B. A. W., E. D. Supervision: A. W., S. M., P. J. Funding acquisition: P. J., C. V., A. W., E. D.

## Conflicts of interest

B. A. W. and E. D. are paid employees of TissUse GmbH. The remaining authors declare no competing interests.

## Supplementary Material

LC-026-D6LC00362A-s001

## Data Availability

TempO-Seq data used in this study have been registered to BioStudies (accession number: S-RHER381). Gene sets used for GSEA/GSVA based on the studies by Lake *et al.* (2019)^45^ and Aizarani *et al.* (2019)^46^ can be found on MSigDB under Human collections, C8: https://www.gsea-msigdb.org/gsea/msigdb/human/genesets.jsp?collection=C8. Links to the gene sets on MsigDB are provided below. Kidney sets: https://www.gsea-msigdb.org/gsea/msigdb/human/geneset/LAKE_ADULT_KIDNEY_C3_PROXIMAL_TUBULE_EPITHELIAL_CELLS_S1_S2.html; https://www.gsea-msigdb.org/gsea/msigdb/human/geneset/LAKE_ADULT_KIDNEY_C4_PROXIMAL_TUBULE_EPITHELIAL_CELLS_S2.html; https://www.gsea-msigdb.org/gsea/msigdb/human/geneset/LAKE_ADULT_KIDNEY_C7_PROXIMAL_TUBULE_EPITHELIAL_CELLS_S3.html Liver sets: https://www.gsea-msigdb.org/gsea/msigdb/human/geneset/AIZARANI_LIVER_C11_HEPATOCYTES_1.html; https://www.gsea-msigdb.org/gsea/msigdb/human/geneset/AIZARANI_LIVER_C14_HEPATOCYTES_2.html; https://www.gsea-msigdb.org/gsea/msigdb/human/geneset/AIZARANI_LIVER_C17_HEPATOCYTES_3.html; https://www.gsea-msigdb.org/gsea/msigdb/human/geneset/AIZARANI_LIVER_C30_HEPATOCYTES_4.html. Any additional supporting data are available from the corresponding author upon reasonable request. Supplementary information (SI) is available. See DOI: https://doi.org/10.1039/d6lc00362a.
