# Supplementary material for: A human iPSC-derived kidney–liver organ-on-a-chip platform for modeling inter-organ crosstalk
Source: Lab Chip. 2026 Aug 13;26(19):5311–26. doi: 10.1039/d6lc00362a (PMC13519871; doi:10.1039/d6lc00362a)
Supplement: Supplementary file 1 [file LC-026-D6LC00362A-s001.pdf]

Supplementary Information (SI) for Lab on a Chip.
This journal is © The Royal Society of Chemistry

Supplementary materials

Table S1: STR profile of SIGi001-A.

STR	SIGi001-A
D8S1179	10,14
D21S11	28,30
D7S820	8,11
CSF1PO	10,12
D3S1358	15,18
TH01	9,9.3
D13S317	8,12
D16S539	11,12
D2S1338	17,20
D19S433	13,13
vWA	16,16
TPOX	8,9
D18S51	13,16
AMEL	X,X
D5S818	12,13
FGA	22,25

Table S2: Composition of liver differentiation media (LDM).

Media component	Supplier	Catalog number	Final concentration	Total in 500 mL
DMEM-LG	Gibco	31885	57%	286.8 mL
MCDB (in 1L of H_2O)	Sigma	M6670	40%	200 mL
Penicillin/streptomycin	Sigma	P4333	-	5 mL
L-ascorbic acid (in H_2O)	Sigma	A8960	0.1 mM	5 mL
Linoleic acid- BSA	Sigma	L9530	-	1.25 mL
Insulin, transferrin, sodium selenium (ITS)	Gibco	4140-045	-	1.25 mL
B-Mercaptoethanol	Gibco	31350	-	0.5 mL
Dexamethasone (in H_2O)	Sigma	D-2915	1 µM	100 µL

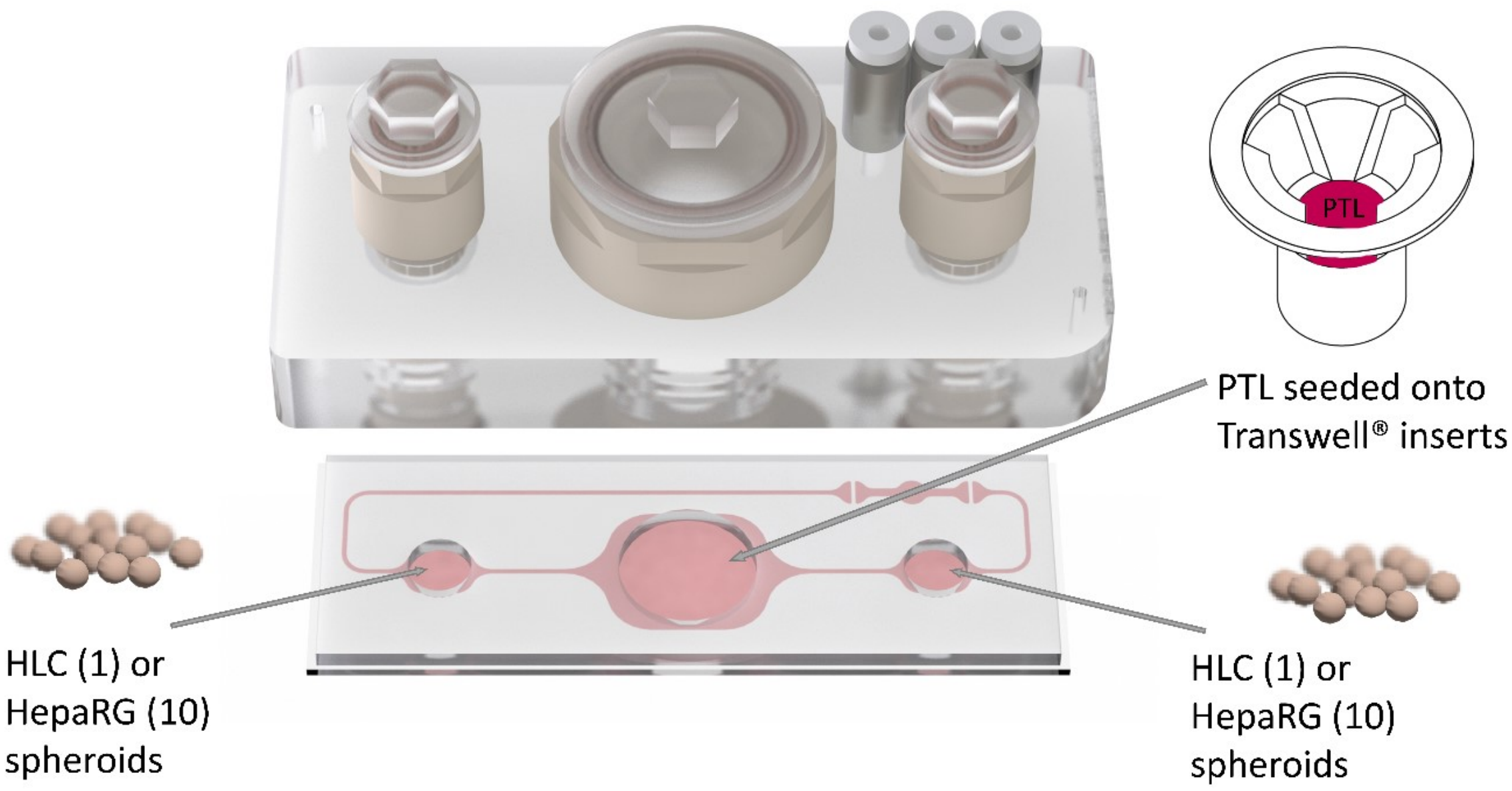

Figure S1: Schematic overview of the TissUse HUMIMIC chip-3 and which cell types are cultured in each compartment.

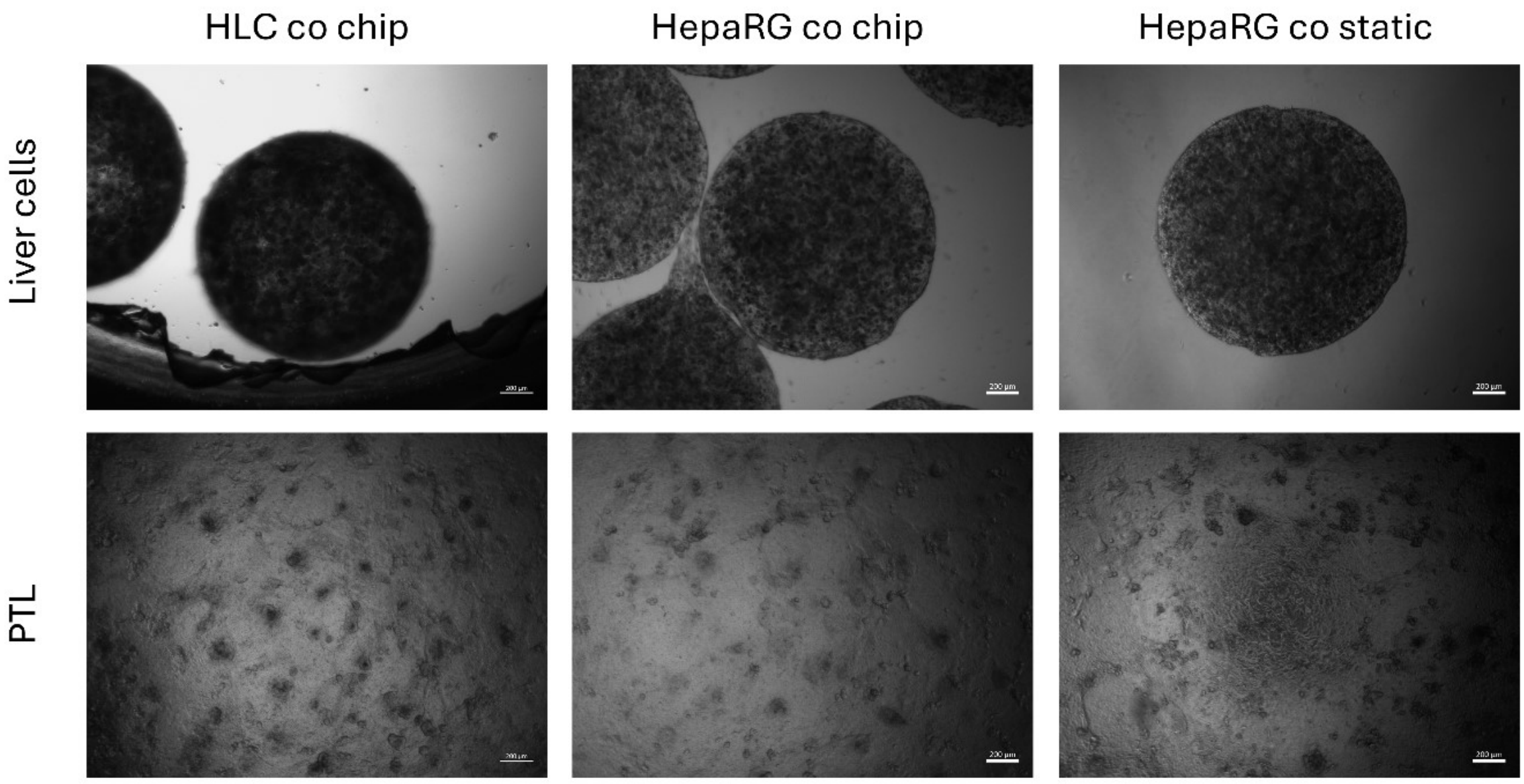

Figure S2: Representative phase contrast images of HLC-PTL co-cultures and HepaRG-PTL co-cultures. Pictures were taken on day 4 of co-culture. Scale bar = 200 μm.

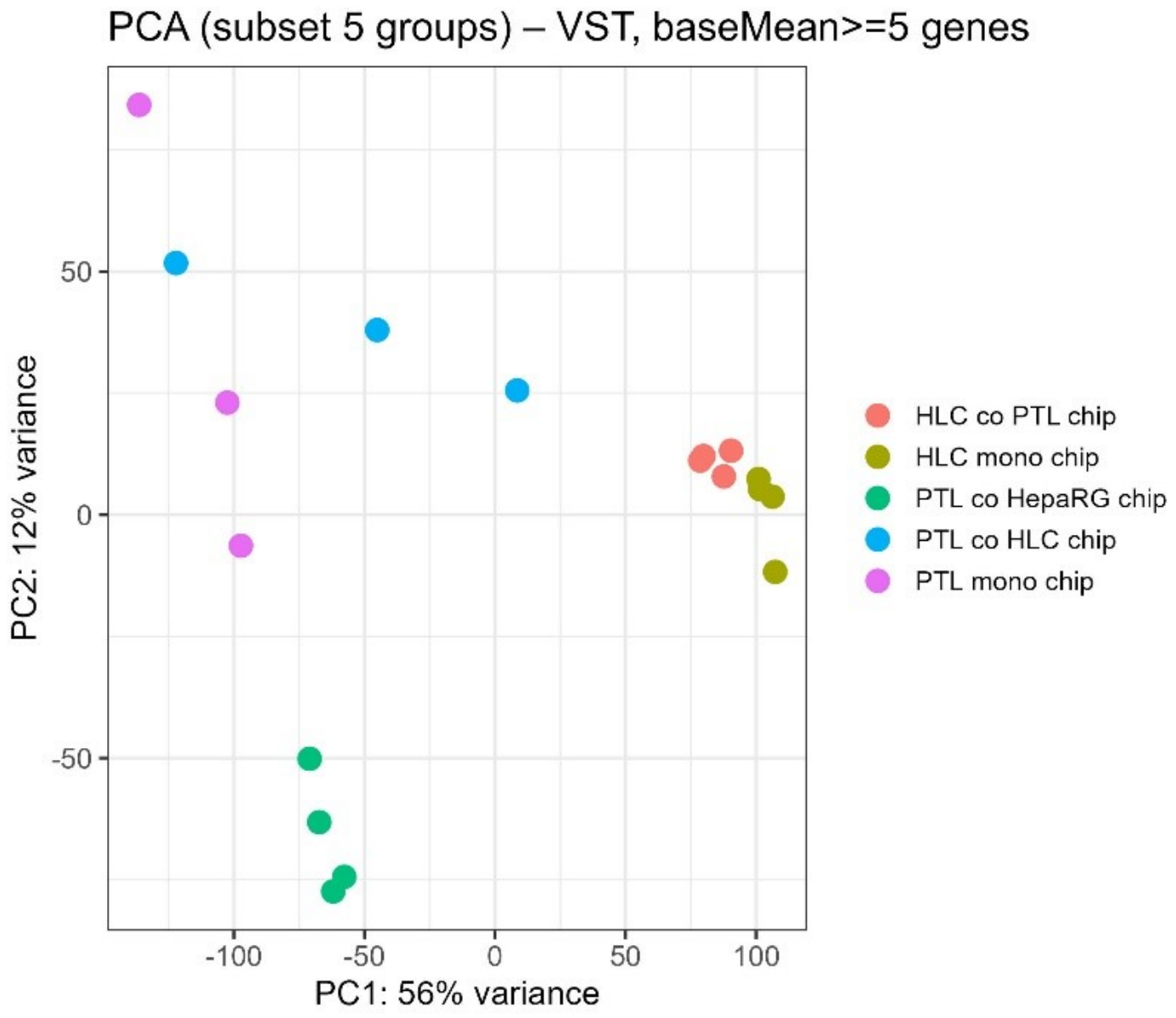

Figure S3: Principal component analysis (PCA) of HLC organobodies and PTL maintained in mono- and co-cultures.

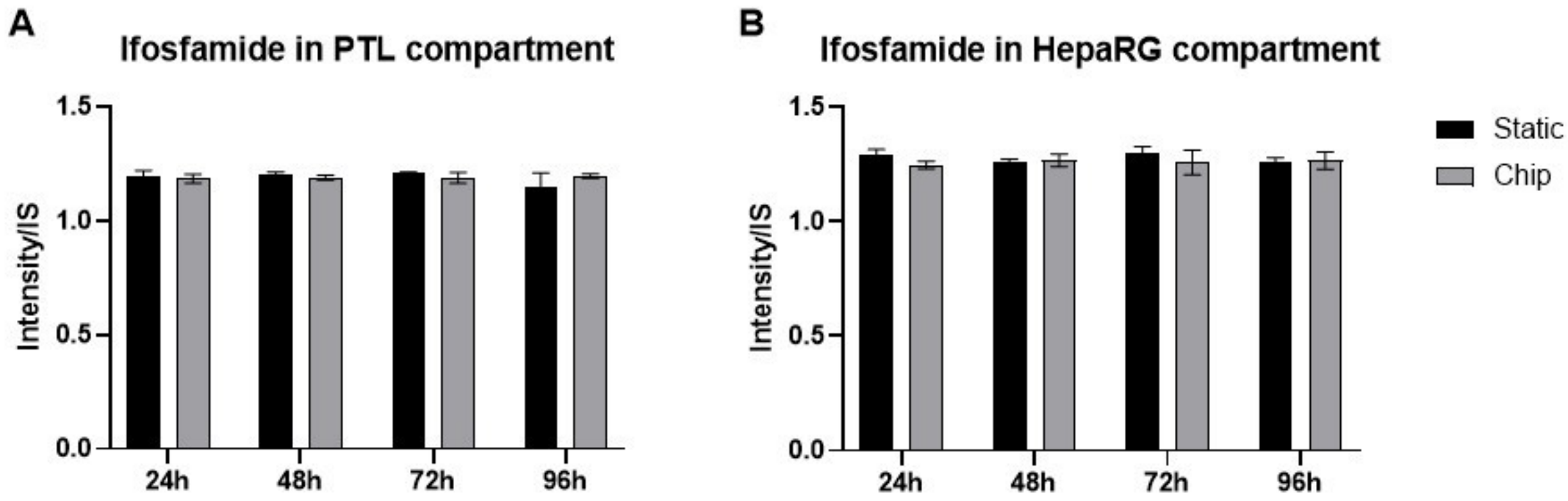

Figure S4: Intensity/internal standard (IS) of ifosfamide in PTL-HepaRG co-cultures.
(A) Intensity/IS of ifosfamide in the apical compartment containing PTL.
(B) Intensity/IS of ifosfamide in the basolateral compartment containing HepaRG spheroids
Error bars depict SD; data is based on n=5 per condition.

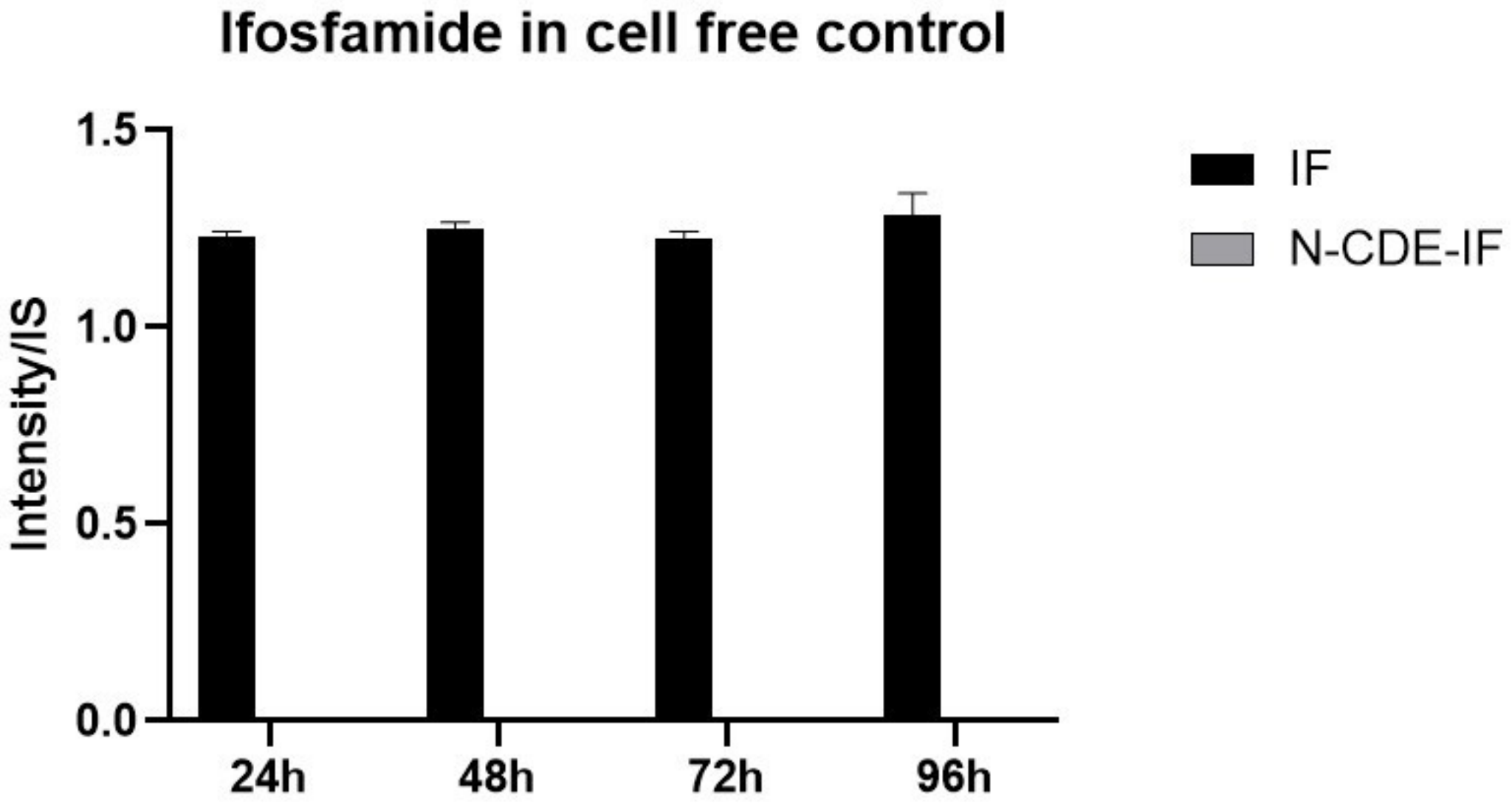

Figure S5: Intensity/internal standard (IS) of ifosfamide (IF) and N-dechloroethyl ifosfamide (N-DCE-IF) in cell free static control treated with ifosfamide (IF) to assess plastic binding, related to figure 5. Error bars depict SD; data is based on n=4 per condition

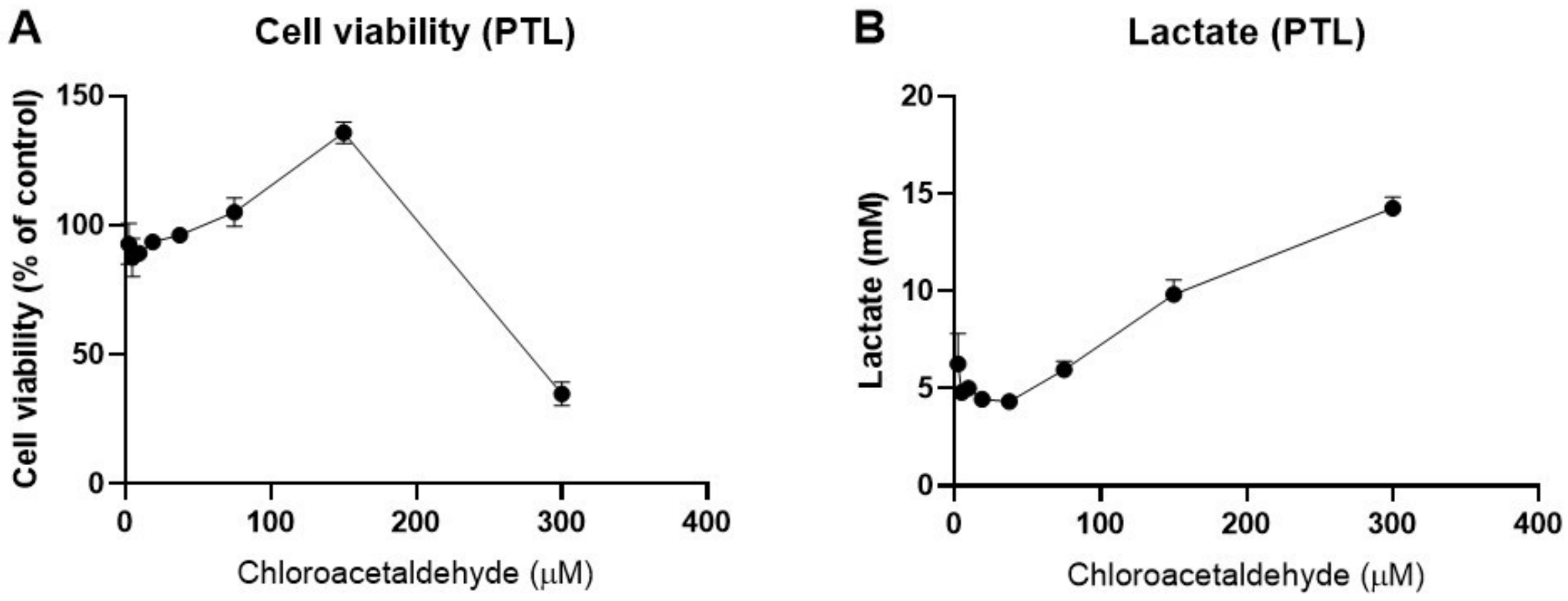

Figure S6: Effects of chloroacetaldehyde (CAA) on PTL mono-cultures after 24 hours of treatment.

(A) Cell viability after treatment with CAA in PTL mono-cultures
(B) Lactate concentration in the media after treatment with CAA in PTL mono-cultures, as a measure of cellular stress.

Error bars depict SD, data is based on n=3 per condition
